# Traditional Chinese Medicine for Post-Stroke Cognitive Impairment: A Systematic Review and Meta-Analysis

**DOI:** 10.3389/fphar.2022.816333

**Published:** 2022-02-14

**Authors:** Wei Shen, Xueming Fan, Liuding Wang, Yunling Zhang

**Affiliations:** Xiyuan Hospital, China Academy of Chinese Medical Sciences, Beijing, China

**Keywords:** traditional Chinese medicine, post-stroke cognitive impairment, systematic review, cognitive function, potential mechanism

## Abstract

**Background:** Post-stroke cognitive impairment (PSCI) affects more than one-third of stroke patients, and causes much greater harm to long-term function than the initial brain damage. No conventional Western medications have shown convincing clinical effectiveness for treating PSCI. Research shows that Traditional Chinese medicine (TCM) can improve cognitive function in patients. However, the clinical efficacy and safety remain controversial. The aim of this study was to examine the effectiveness and harmful effects of TCMs in the treatment of PSCI.

**Method:** We searched seven databases and two clinical registration websites for randomized controlled trials (RCTs). The revised Cochrane risk of bias tool (RoB 2.0) was used to evaluate the methodological quality and RevMan 5.4 was used for data analysis. This study has been submitted to PROSPERO with registration number is CRD42020149299.

**Results:** We included 34 studies in this review. The results of this study showed that TCM adjuvant therapy improved scores on the MoCA [MD = 2.55, 95% CI (1.56, 3.53), *p* < 0.00001; MD = 3.07, 95% CI (1.98, 4.17), *p* < 0.00001 at treatment duration of <3 and 3 months, respectively], MMSE [MD = 2.55, 95% CI (1.99, 3.10), *p* < 0.00001; MD = 2.53, 95% CI (1.59, 3.47), *p* < 0.00001; MD = 2.91, 95% CI (1.26, 4.56), *p* = 0.0006; MD = 3.11, 95% CI (−0.04, 6.27), *p* = 0.05 at treatment duration of <3, 3, 4, and 6 months, respectively], and BI [MD = 7.34, 95% CI (3.83, 10.85), *p* < 0.0001; MD = 8.98, 95% CI (4.76, 13.21), *p* < 0.0001 at treatment duration of <3 and 3 months, respectively] and reduced scores on the ADL (MD = −8.64, 95% CI (−9.83, −7.45), *p* < 0.00001; MD = −2.00, 95% CI (−2.94, −1.06), *p* < 0.0001 at treatment duration of 3 and 4 months, respectively], NIHSS [MD = −2.48, 95% CI (−4.97, 0.00), *p* = 0.05; MD = −3.81, 95% CI (−6.21, −1.40), *p* = 0.002 at treatment duration of <3 and 3 months, respectively], and CSS [MD = −2.47, 95% CI (−3.49, −1.45), *p* < 0.00001 at a treatment duration of 3 months]. No serious adverse reactions were observed.

**Conclusion:** Despite the significant positive results, the present evidence supports, to a limited extent because of the methodological flaws and herbal heterogeneity, that TCM adjuvant therapy can be used for patients with PSCI. While, further rigorous RCTs are warranted to confirm the efficacy and safety of TCM.

**Systematic Review Registration:** https://www.crd.york.ac.uk/prospero, identifier CRD42020149299.

## Introduction

Post-stroke cognitive impairment (PSCI) is one of the main complications of stroke (Mijajlović et al., 2017), and causes more harm to long-term function than the initial brain injury (Swartz et al., 2016). PSCI can be categorized as post-stroke cognitive impairment no dementia (PSCIND) and post-stroke dementia (PSD), according to the severity of cognitive decline. Previous research indicates that the overall prevalence of PSCI varies from 30% to 50% (Mellon et al., 2015), and the incidence of dementia in patients with severe and minor stroke is 34.4% and 8.2%, respectively (Pendlebury and Rothwell, 2019). PSCI frequently causes severe damage to attention and executive ability (Aam et al., 2020; Zhang and Bi, 2020), affects activities of daily living (Blomgren et al., 2019), and places heavy financial burdens on paramedics and medical institutions (Black et al., 2018). On 4 May 2016, the American Heart Association and the American Stroke Association jointly issued the Guidelines for Adult Stroke Rehabilitation and Recovery, which emphasize the importance of memory and cognitive assessment in post-stroke recuperation. For example, it is recommended that stroke patients receive cognitive function training for post-acute recovery (Class I, Level A evidence), and that all stroke patients should be screened for cognitive deficits before discharge (Class I, Level B evidence) (Gittler and Davis, 2018). Although over one-third of stroke patients may develop PSCI, its underlying pathogenesis remains unclear (Kim et al., 2020a). Various mechanisms may play an important role in PSCI, such as cerebral small vessel disease (Teng et al., 2017), lesions in neuroanatomical structures (Sun et al., 2014), neuroinflammation and oxidative stress (Zhang et al., 2021). Recent study indicated that endovascular treatment can result in better cognitive performance of stroke patients (Lattanzi et al., 2020), while there is still a lack of sufficient evidence to support oral drug therapy in this process (Quinn et al., 2021). Expert consensus recommends the use of drugs for the treatment of vascular cognitive impairment and Alzheimer’s disease (Dong et al., 2017; Wang et al., 2021a); however, the restoration of cognitive function or prevention of further decline after stroke are still uncertain (Brainin et al., 2015). Therefore, identification of possible specific treatments plays an important role in the prognosis of PSCI (Wang, 2020a).

In Asia, Traditional Chinese medicine (TCM) is widely used to improve the quality of life of patients with neurodegenerative disease and neuropsychiatric disorders (Feng et al., 2021). Previous research has shown that TCM plays an important role in improving cognitive function in patients. For instance, Danshen extract reduces the contents of tumor necrosis factor-α, interleukin-1β, and interleukin-6 in the hippocampus and facilitates learning and memory in rats (Kim et al., 2015); huperzine A, an extract from *Huperzia serrata*, selectively inhibits acetylcholinesterase activity, increases acetylcholine levels in the brain, and improves cognitive function in patients with dementia (Xu et al., 2012; Xing et al., 2014); berberine effectively alleviates amyloid-β-induced neuroinflammation and regulates amyloid precursor protein metabolism (Jia et al., 2012); and tetramethylpyrazine inhibits angiogenesis and platelet aggregation and improves cerebral microcirculation (Cai et al., 2014; Cui et al., 2015). TCM may be a useful drug therapy to improve cognitive function, but small sample sizes, lack of long-term follow-up, and heterogeneity between studies (Dong et al., 2017) mean that the clinical efficacy and safety of TCM remain controversial. A previous systematic review demonstrated that TCM has a positive effect on cognitive function in PSCI with no serious adverse events (Shen et al., 2020). However, the search strategy of this study was incomplete and several new controlled trials have been conducted since its publication. Therefore, an updated systematic review of the use of TCM for PSCI is needed to provide a firm basis for the treatment of this disease.

## Methods

### Protocol Registration

This systematic review was registered with the International Prospective Register of Systematic Reviews (PROSPERO) (ID = CRD42020149299).

### Search Methods

A comprehensive retrieval was conducted using the following medical databases: China National Knowledge Infrastructure (CNKI), Wanfang Database, China Science and Technology Journal Database (VIP), and Chinese Biomedical literature Service System (SinoMed) in Chinese, and PubMed, EMBASE, and Cochrane Library in English. The retrieval time was from database inception to February 2021. The search strategy included a combination of medical subject headings and free-text terms. Ongoing or unpublished studies registered on the clinical registration websites (e.g., Chinese Clinical Trial Registry, Clinical Trials.gov) were also searched to obtain more general empirical data. In October 2021, we updated the databases search using the same search method.

### Inclusion Criteria

#### Type of Study

Randomized controlled trials (RCTs) that evaluated the clinical effectiveness and safety of TCM for PSCI were eligible. Studies had to be full-text articles in English or Chinese, regardless of study site, publication date, or study status.

### Type of Participant

We included studies in which patients had a firm diagnosis of PSCI (Wang, 2020a). In addition, because PSCI suggests a causal relationship between stroke and cognitive decline, studies that included a first diagnosis of stroke and a second diagnosis of cognitive impairment were also included.

### Type of Intervention

Included studies featured an intervention group treated with TCM combined with conventional Western medication and a control group that received the same conventional Western medication. TCM interventions were defined as prescriptions containing multiple herbs, a single herb, or Chinese patent medicines, with no restrictions on dosage, dosage form, and mode of administration. Conventional Western medication had to feature expert consensus recommended drugs (Dong et al., 2017; Wang, 2020a; Wang et al., 2021a), such as Memantine, Nimodipine, and Oxiracetam. We placed no restriction on the treatment course, but the intervention and control groups had to be the same across studies.

### Type of Outcome

The primary outcome was cognitive assessment using at least one of the internationally recognized assessment scales, such as the Montreal Cognitive Assessment (MoCA), Mini-Mental State Examination (MMSE), Alzheimer’s Disease Assessment Scale-Cognitive Subscale (ADAS-cog), Vascular Dementia Assessment Scale-Cognitive Subscale (VaDAS-cog), Wechsler Memory Scale (WMS), Hasegawa Dementia Scale (HDS), or Clinical Dementia Rating (CDR).

The secondary outcomes comprised assessment of activities of daily living [using for example the Barthel Index (BI) or the Activities of Daily Living Scale (ADL)] and assessment of neurological deficits [using for example the National Institutes of Health Stroke Scale (NIHSS) or the China Stroke Scale (CSS)].

Safety indicators, such as the incidence of adverse events or adverse reactions, were used to analyze the clinical safety.

### Exclusion Criteria

Studies that used folk medicine and Chinese medicine extracts were excluded, because they were not considered as TCM. Studies that did not clearly identify the drug use or that had incomplete or duplicated data were excluded as well.

### Study Selection and Data Extraction

Endnote X9 (Clarivate Analytics, https://www.endnote.com) was used to manage the literatures. The titles, abstracts, and full text of potentially relevant studies were read and eligible studies were identified. The reasons for exclusion were recorded. Two authors independently conducted the literature screening and information extraction according to a predetermined standardized information extraction table. The data items comprised 1) General information (title, first author, publication date); 2) Study characteristics (sample size, method of randomization, allocation); 3) Participant characteristics (age, gender, course of disease); 4) Intervention characteristics (intervention measures, course of treatment, composition of TCM, follow-ups); 5) Outcomes (outcome measures, adverse events). A third author resolved any disagreements.

### Risk of Bias Assessment of Included Studies

We assessed the methodological quality of the included studies using the revised Cochrane risk of bias tool (RoB 2.0) (Sterne et al., 2019). The evaluation domains comprised randomization process, deviations from intended interventions, missing outcome data, measurement of the outcome, and selection of the reported result. The risk of bias for each domain was evaluated as high risk, low risk and some concerns. Results of the risk of bias assessment were summarized using a risk of bias graph and a risk of bias summary figure. Two authors independently extracted the information. A third author resolved any disagreements.

### Data Synthesis and Statistical Analysis

RevMan 5.4 from the Cochrane Collaboration was used for data analysis. We used the pooled relative risk [and confidence intervals (CI)] to analyze dichotomous outcomes and the mean difference (MD) or standard mean difference (SMD) (and CI) to analyze continuous outcomes. For some studies that reported only the median, minimum and maximum values, and/or the first and third quartiles, we used a unified approach to estimate the sample mean and standard deviation from these values [Method 1 (Wan et al., 2014; Luo et al., 2018) for normally distributed data, Method 2 (Mcgrath et al., 2020) for non-normally distributed data]. Cochrane’s *X* ^2^ test and *I* ^2^ were used to assess heterogeneity. A fixed-effect model was used for meta-analysis if the statistical heterogeneity among the results was not obvious (*p* > 0.1, *I* ^2^ < 50%), and a random-effect model was used for meta-analysis if the statistical heterogeneity among the results was obvious (*p* ≤ 0.1, 50% ≤ *I* ^2^ < 90%). Descriptive analysis was used to summarize the outcomes if the statistical heterogeneity between the results was significant (*p* ≤ 0.1, *I* ^2^ ≥ 90%). If the treatment course had a substantial clinical effect, we carried out subgroup analysis to examine differences in the length of therapy. Publication bias was examined using funnel plot analyses if the number of studies was ≥10.

### Certainty Assessment of Evidence

The certainty of evidence for each specific outcome was evaluated by using the Grading of Recommendations, Assessment, Development, and Evaluations (GRADE) system (Guyatt et al., 2008). Two authors separately assessed the quality of outcome evidence as high, moderate, low, or very low, which can be downgraded for five reasons (risk of bias, imprecision, inconsistency, indirectness, and publication bias) and upgraded for three reasons (large magnitude of an effect, dose-response gradient, and effect of plausible residual confounding).

## Results

### Study Selection

**FIGURE 1:**
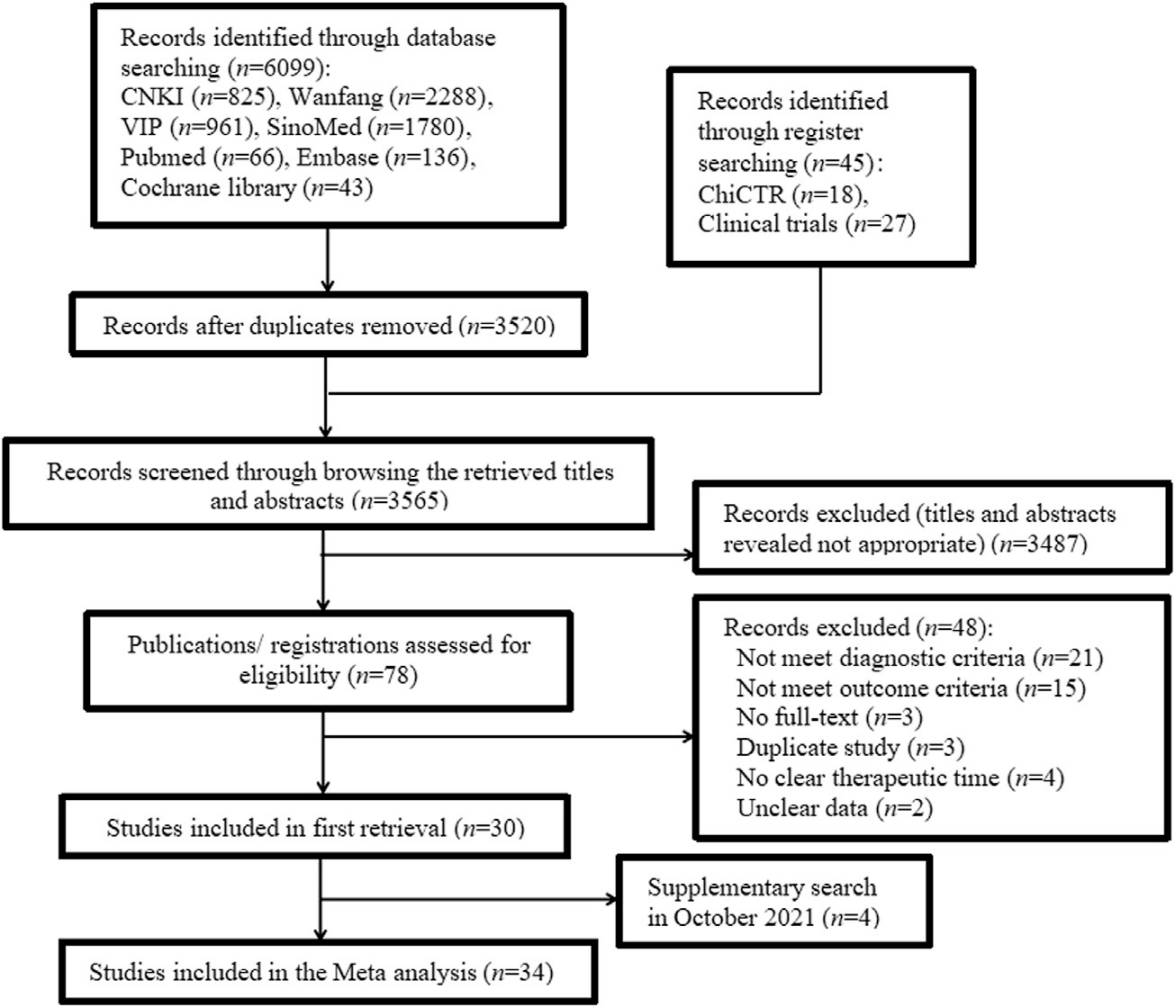
Flowchart of study inclusion.

The search strategy retrieved 6,144 studies: 6,099 were identified using database searches and 45 identified using register searches (Supplementary Table S1). After removing 2,579 duplicates from database sources, we screened the retrieved titles and abstracts and excluded 3,487 studies. Of the remaining 78 studies, 48 were excluded because they did not meet the diagnostic or outcome criteria, were not full-text articles, were duplicate studies, did not clearly specify therapy duration, or contained unclear data (Supplementary Table S2). In October 2021, we supplemented the retrieval of four studies that met the inclusion criteria. Finally, 34 studies (Liu and Liu, 2005; Mo, 2010; Cheng et al., 2012; Liu, 2013; Liu, 2014; Xu et al., 2014; Liu, 2015; Luo and Liu, 2016; Ma and Lin, 2016; Tan et al., 2016; Zhuo et al., 2016; Feng et al., 2017; Jiang et al., 2018; Liu, 2018; Ren et al., 2018; Song, 2018; Wang et al., 2018; Huang et al., 2019a; Huang et al., 2019b; Huang, 2019; Liu, 2019; Xu et al., 2019; You, 2019; Wang, 2020b; Chen et al., 2020; Han, 2020; Liu, 2020; Liu et al., 2020; Ma and Zhou, 2020; Meng et al., 2020; Wang et al., 2021b; Li, 2021; Liu and Yuan, 2021; Yang et al., 2021) were included. The screening process is summarized in the flow diagram in Figure 1.

### Study Inclusion Characteristics

We included 34 studies in this review. All studies were published in Chinese and conducted between 2005 and 2021. Of the included studies, four studies (Liu and Liu, 2005; Jiang et al., 2018; Liu, 2019; Meng et al., 2020) provided data on PSD and ten studies (Mo, 2010; Xu et al., 2014; Luo and Liu, 2016; Ren et al., 2018; Wang et al., 2018; You, 2019; Han, 2020; Liu et al., 2020; Ma and Zhou, 2020; Yang et al., 2021) provided data on PSCIND. The sample size of the included trials varied from 40 to 122 participants, aged 45–85 years. The course of treatment ranged from 0.5 to 6 months. A total of 26 studies used the MMSE as the primary outcome measure and 20 studies used the MoCA. Details of the exposure and outcomes of each included study are summarized in Table 1.

**TABLE 1:** Characteristics of included studies.

Study ID	Sample size		Course of disease (month)		Mean age (year)		Male/Female (Male%)		Intervention		Course of treatment (month)	Outcomes
	Trial	Control	Trial	Control	Trial	Control	Trial	Control	Trial	Control		
Meng et al. (2020)	42	42	5.11 ± 1.27	5.07 ± 1.35	67.49 ± 4.93	68.54 ± 3.64	25/17 (60%)	23/19 (55%)	Peiyuan Tongnao capsule + Oxiracetam	Oxiracetam	3	(2) (4) (5) (7)
Liu (2019)	46	46	7.21 ± 1.02	7.15 ± 0.96	62.95 ± 2.68	63.02 ± 2.74	23/23 (50%)	21/25 (46%)	Yishen Huayu Tongluo decoction + Nimodipine + Piracetam	Nimodipine + Piracetam	6	(2) (7)
Jiang et al., 2018)	40	40	2.91 ± 1.07	2.45 ± 1.21	66.29 ± 4.11	65.39 ± 2.87	21/19 (53%)	23/17 (58%)	Tongnaoling granule + Donepezil	Donepezil	6	(2) (7)
Liu and Liu (2005)	60	60	7 days-3	7 days-3.5	55–81	78–58	34/26 (57%)	38/22 (63%)	TCM decoction + Nimodipine	Nimodipine	1	(2)
Yang et al. (2021)	27	27	0.76 ± 0.47 years	0.69 ± 0.42 years	61.10 ± 15.83	60.82 ± 16.10	15/12 (56%)	16/11 (59%)	Xiongqi Xingnao granule + Butylphthalide	Butylphthalide	2	(1) (2)
Liu et al. (2020)	30	30	12.56 ± 2.23 h	12.48 ± 2.45 h	61.12 ± 6.21	61.35 ± 6.12	15/15 (50%)	17/13 (57%)	Tongqiao Huoxue decoction + Citicoline	Citicoline	0.5	(1) (5) (7)
Ma and Zhou (2020)	47	47	NR	NR	64.2 ± 12.5	63.8 ± 12.4	26/21 (55%)	28/19 (60%)	Tongnao Yisui decoction + Donepezil	Donepezil	3	(2)
Han (2020)	41	43	3.59 ± 1.48	3.23 ± 1.27	56.77 ± 4.67	67.63 ± 3.86	20/21 (49%)	19/24 (46%)	Bushen Yizhi decoction + Donepezil	Donepezil	3	(2) (3) (7)
You (2019)	30	30	NR	NR	63.47 ± 8.21	63.60 ± 7.75	15/15 (50%)	13/17 (43%)	Yiqi Shuxue Tongmai decoction + Donepezil	Donepezil	3	(1) (7)
Ren et al. (2018)	40	40	3.73 ± 0.56	3.82 ± 0.60	67.02 ± 5.38	67.26 ± 5.63	22/18 (55%)	24/16 (60%)	Yishen Huayu decoction + Donepezil	Donepezil	3	(1)
Wang et al. (2018)	30	30	2.8 ± 0.6	3.1 ± 0.5	62.73 ± 9.40	59.53 ± 9.90	14/16 (47%)	19/11 (63%)	Qilong Yizhi granule + Nimodipine	Nimodipine	6	(2) (7)
Luo and Liu (2016)	30	30	2.9 ± 0.6	3.0 ± 0.4	60.25 ± 8.55	62.50 ± 8.40	17/13 (57%)	14/16 (47%)	Modified Ditan decoction + Nimodipine	Nimodipine	3	(2) (3) (5) (7)
Xu et al. (2014)	30	30	3.9 ± 0.6	4.0 ± 0.4	67.5 ± 3.05	68.3 ± 3.2	19/11 (63%)	20/10 (67%)	Qiangli Zengzhi decoction + Nimodipine	Nimodipine	3	(1) (2) (7)
Mo (2010),^a^	30	30	NR	NR	69.6 ± 9.6	72.6 ± 8.5	21/9 (70%)	20/10 (67%)	Ziyin Jiannao tablet + Nimodipine	Nimodipine	2	(2) (6)
Li (2021)	44	44	1.01 ± 0.38 years	1.03 ± 0.31 years	66.57 ± 4.48	66.14 ± 4.53	23/21 (52%)	24/20 (55%)	TCM decoction + Nimodipine	Nimodipine	3	(1) (3) (7)
Liu and Yuan (2021)	47	47	14.52 ± 2.79 days	14.35 ± 2.68 days	62.73 ± 7.06	62.15 ± 6.79	27/20 (57%)	26/21 (55%)	Huoxue Huayu Xingnao decoction + Nimodipine	Nimodipine	1	(2) (5)
Wang et al. (2021b)	61	61	19.56 ± 3.25 days	18.98 ± 4.38 days	68.79 ± 5.42	69.23 ± 4.79	39/22 (64%)	37/24 (61%)	Chaimu Xingnao decoction + Oxiracetam	Oxiracetam	1	(1) (2) (3) (7)
Chen et al. (2020)	40	40	1.28 ± 1.12 weeks	1.29 ± 1.08 weeks	65.52 ± 3.94	65.59 ± 3.91	22/18 (55%)	23/17 (58%)	Buyang Huanwu decoction + Aniracetam	Aniracetam	1	(1) (2) (7)
Liu (2020)	30	30	NR	NR	70.03 ± 8.79	67.77 ± 10.51	18/12 (60%)	16/14 (53%)	Shenrong Tongmai capsule + Donepezil	Donepezil	3	(1) (2) (3) (7)
Wang (2020b)	37	37	NR	NR	60.54 ± 11.99	62.54 ± 11.49	17/20 (46%)	22/15 (59%)	Zhongfeng Xingnao liquid + Donepezil	Donepezil	1	(1) (2) (3) (5) (7)
Xu et al. (2019)	30	30	NR	NR	NR	NR	NR	NR	Modified Qufeng Tongqiao decoction + Donepezil	Donepezil	1.5	(1) (2) (7)
Huang et al. (2019b)	41	39	2.68 ± 1.75 years	3.12 ± 1.34 years	68.50 ± 4.90	65.18 ± 5.20	23/18 (56%)	21/18 (54%)	Modified Wuzi Yanzong pill + Donepezil	Donepezil	4	(2) (4) (7)
Huang et al. (2019a)	47	47	NR	NR	63.56 ± 2.24	63.21 ± 2.19	25/22 (53%)	26/21 (55%)	Buchang Naoxintong capsule + Donepezil	Donepezil	3	(1) (2) (3) (5) (7)
Huang (2019)	27	30	NR	NR	71.00 (69.00,74.00)	71.50 (69.00,74.00)	21/6 (78%)	20/10 (67%)	Zuogui decoction + Donepezil	Donepezil	3	(1) (2) (7)
Liu (2018)	40	40	NR	NR	68.35 ± 9.76	70.35 ± 9.75	28/12 (70%)	25/15 (62%)	Tongnao decoction + Donepezil	Donepezil	3	(2) (3) (5) (7)
Song (2018)	30	30	NR	NR	NR	NR	19/11 (63%)	16/14 (53%)	Shuxue Tongmai granule + Donepezil	Donepezil	3	(1) (2) (3) (6) (7)
Feng et al. (2017)	50	50	4.6 ± 2.5 years	4.5 ± 2.4 years	68.2 ± 11.3	67.8 ± 10.8	30/20 (60%)	32/18 (64%)	Huannao Yicong decoction + Piracetam + Donepezil	Piracetam + Donepezil	3	(1) (2) (3) (7)
Ma and Lin, 2016)	60	60	NR	NR	56.5 ± 6.3	57.4 ± 6.7	39/21 (65%)	36/24 (60%)	Dengzhan Shengmai capsule + Donepezil	Donepezil	3	(1) (3) (7)
Tan et al. (2016),^b^	40	40	NR	NR	60.1 ± 4.4	59.2 ± 6.3	21/19 (53%)	23/17 (58%)	Shuxue Tongmai granule + Donepezil	Donepezil	3	(1) (3) (6)
Zhuo et al. (2016)	53	53	NR	NR	69.9 ± 7.8	70.2 ± 7.3	27/26 (51%)	29/24 (55%)	Yangxue Qingnao granule + Butylphthalide	Butylphthalide	1	(1) (2)
Liu (2015)	42	42	NR	NR	69.9 ± 7.2	70.5 ± 7.0	24/18 (57%)	22/20 (52%)	Buyang Huanwu decoction + Butylphthalide	Butylphthalide	0.5	(1)
Liu (2014),^c^	50	50	NR	NR	58.5 ± 20.4		47/53 (47%)		Tianzhi granule + Oxiracetam	Oxiracetam	1	(2) (4) (7)
Liu (2013)	20	20	NR	NR	70.8 ± 9.0	69.6 ± 9.1	14/6 (70%)	15/5 (75%)	Tongqiao Huoxue decoction + Nimodipine	Nimodipine	1	(1)
Cheng et al. (2012),^c^	44	40	NR	NR	54–80	55–79	26/18 (59%)	24/16 (60%)	Tianzhi granule + Piracetam	Piracetam	1	(2) (4) (7)

Abbreviations: NR, Not Reported; Montreal Cognitive Assessment (MoCA); Mini-Mental State Examination (MMSE); Barthel Index (BI); Activities of Daily Living Scale (ADL); National Institutes of Health Stroke Scale (NIHSS); China Stroke Scale (CSS); Adverse events.

a The CSS, evaluation standard was incorrect, and the data were not included in the study.

b The CSS, data for the two groups before and after treatment were the same; this was considered incorrect and was not included in the study.

c The ADL, data were inconsistent with the description, which was not included in the study.

### Risk of Bias Assessment

**FIGURE 2:**
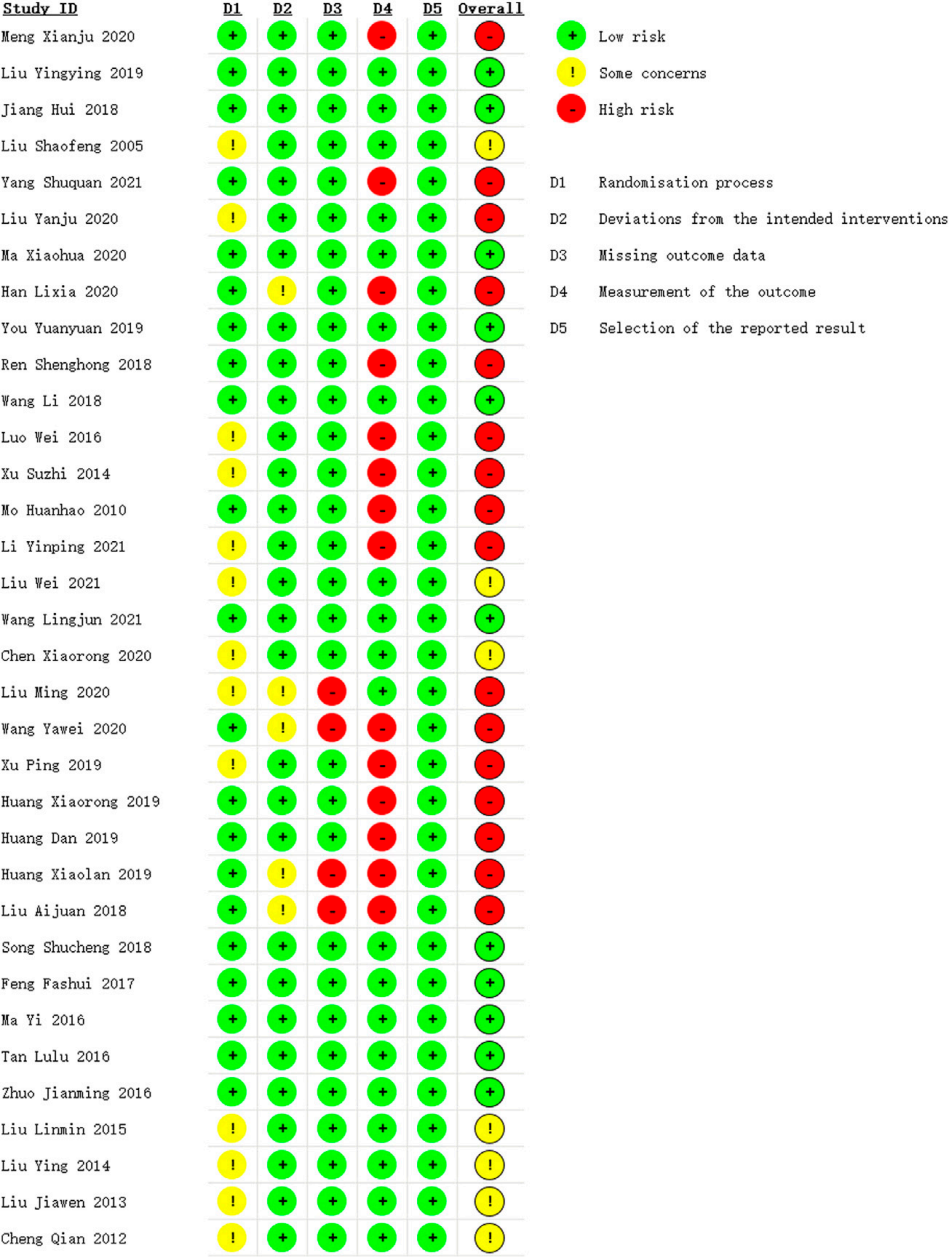
Risk of bias summary.

The overall risk of bias was identified high in sixteen studies (Meng et al., 2020; Yang et al., 2021; Liu et al., 2020; Han, 2020; Ren et al., 2018; Luo and Liu, 2016; Xu et al., 2014; Mo, 2010; Li, 2021; Liu, 2020; Wang, 2020; Xu et al., 2019; Huang et al., 2019b; Huang et al., 2019a; Huang, 2019; Liu, 2018), moderate in seven studies (Liu and Liu, 2005; Liu and Yuan, 2021; Chen et al., 2020; Liu, 2015; Liu, 2014; Liu, 2013; Cheng et al., 2012) and low in eleven studies (Liu, 2019; Jiang et al., 2018; Ma and Zhou, 2020; You, 2019; Wang et al., 2018; Wang et al., 2021b; Song, 2018; Feng et al., 2017; Ma and Lin, 2016; Tan et al., 2016; Zhuo et al., 2016), as is shown in Figure 2 and Supplementary Figure S1. The randomization process was considered some concerns as thirteen studies (Liu and Liu, 2005; Cheng et al., 2012; Liu, 2013; Liu, 2014; Xu et al., 2014; Liu, 2015; Luo and Liu, 2016; Xu et al., 2019; Chen et al., 2020; Liu, 2020; Liu et al., 2020; Li, 2021; Liu and Yuan, 2021) did not provide enough information in the method of random sequence generation. We rated some concerns in the deviations from intended interventions for the use of per-protocol analysis of five studies (Liu, 2018; Huang, 2019; Wang, 2020b; Han, 2020; Liu, 2020), and the missing outcome data of four studies (Liu, 2018; Huang, 2019; Wang, 2020b; Liu, 2020) were showed high risk due to the shedding cases were mostly depend on the clinical efficacy of drugs. The risk of bias in the measurement of the outcome in fourteen studies (Mo, 2010; Xu et al., 2014; Luo and Liu, 2016; Liu, 2018; Ren et al., 2018; Huang et al., 2019a; Huang et al., 2019b; Huang, 2019; Xu et al., 2019; Wang, 2020b; Han, 2020; Meng et al., 2020; Li, 2021; Yang et al., 2021) were evaluated as high because the use of composite index to measure clinical efficacy rate which could not objectively reflect the intervention effect of TCM treatment.

### Cognitive Function Assessment

#### MoCA

**FIGURE 3:**
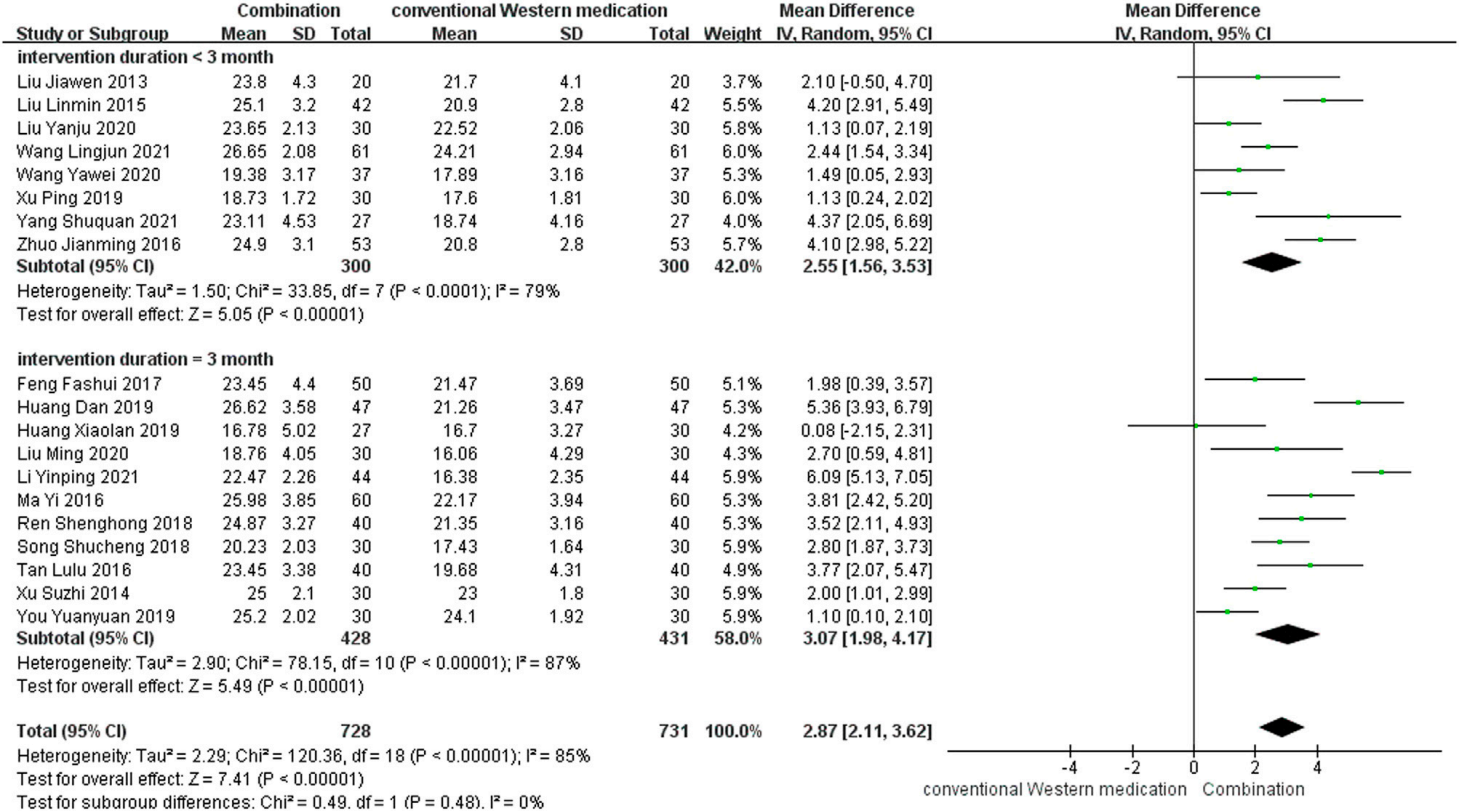
Meta-analysis results for effect of TCM plus conventional Western medication on MoCA.

TCM plus conventional Western medication was used in 20 studies, as shown in Figure 3. Of these studies, one study (Chen et al., 2020) lacked information about post-treatment scores, but the 6 months follow-up results showed that TCM adjuvant therapy significantly improved long-term MoCA scores [MD = 4.14, 95% CI (3.19, 5.09)]. The subgroup analysis of therapy duration showed that eight studies (Liu, 2013; Liu, 2015; Zhuo et al., 2016; Xu et al., 2019; Wang, 2020b; Liu et al., 2020; Wang et al., 2021b; Yang et al., 2021) had a therapy duration of <3 months (*p* < 0.0001, *I* ^2^ = 79%) and eleven studies (Xu et al., 2014; Ma and Lin, 2016; Tan et al., 2016; Feng et al., 2017; Ren et al., 2018; Song, 2018; Huang et al., 2019a; Huang, 2019; You, 2019; Liu, 2020; Li, 2021) had a therapy duration of 3 months (*p* < 0.00001, *I* ^2^ = 87%), suggesting some heterogeneity. The random-effect model showed a significant effect of TCM adjuvant therapy on MoCA scores [MD = 2.55, 95% CI (1.56, 3.53), *p* < 0.00001; MD = 3.07, 95% CI (1.98, 4.17), *p* < 0.00001]. The funnel plot was asymmetrical, indicating the existence of publication bias (Supplementary Figure S2).

#### MMSE

**FIGURE 4:**
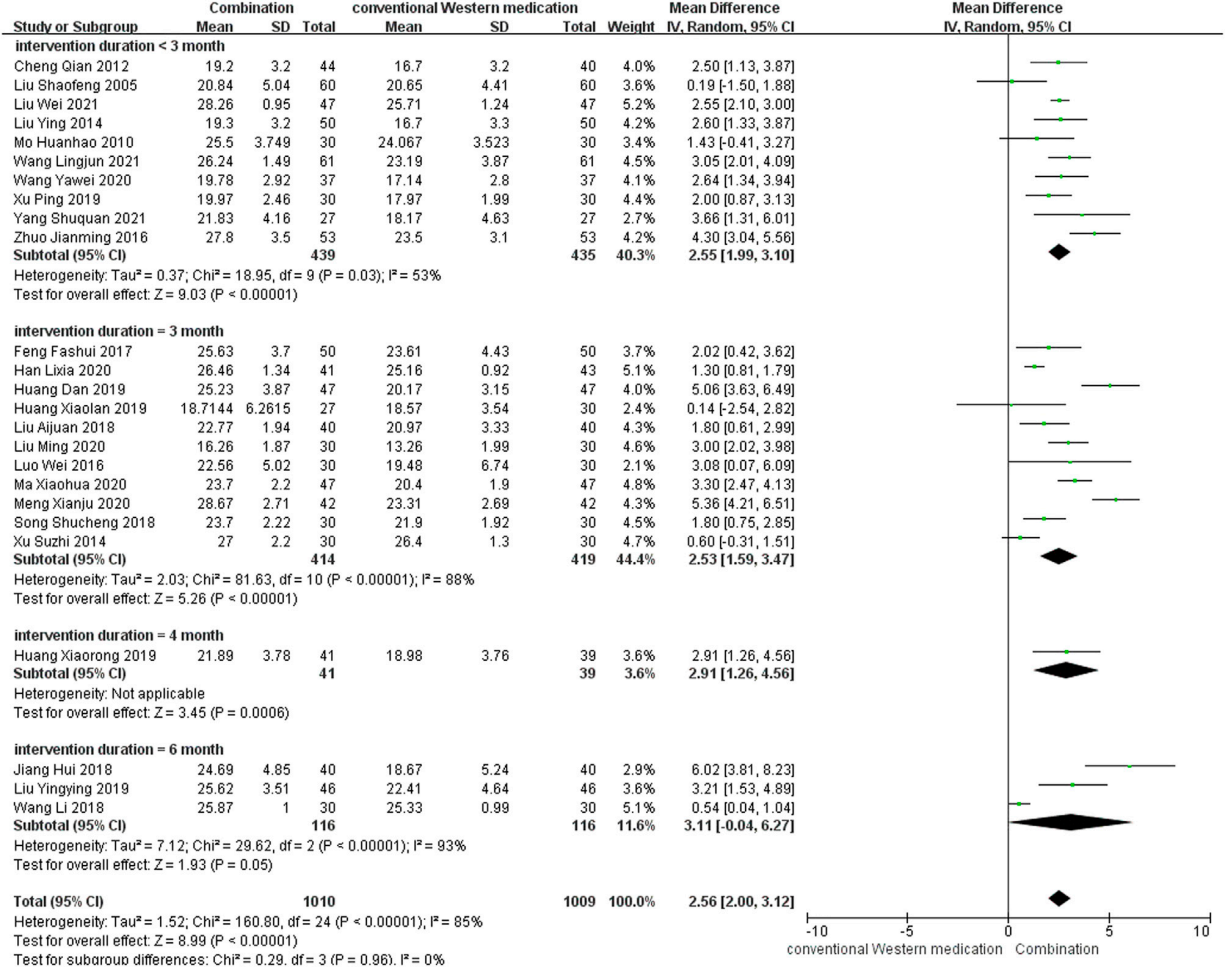
Meta-analysis results for effect of TCM plus conventional Western medication on MMSE.

TCM plus conventional Western medication was used in 26 studies, as shown in Figure 4. Of these, one study (Chen et al., 2020) did not mention post-treatment scores, but the 6 months follow-up results showed that TCM adjuvant therapy had a beneficial effect on long-term MMSE scores [MD = 5.00, 95% CI (3.77, 6.23)]. Subgroup analysis was carried out to examine differences in therapy duration. Ten studies (Liu and Liu, 2005; Mo, 2010; Cheng et al., 2012; Liu, 2014; Zhuo et al., 2016; Xu et al., 2019; Wang, 2020b; Wang et al., 2021b; Liu and Yuan, 2021; Yang et al., 2021) had a therapy duration of <3 months (*p* = 0.03, *I* ^2^ = 53%), eleven studies (Xu et al., 2014; Luo and Liu, 2016; Feng et al., 2017; Liu, 2018; Song, 2018; Huang et al., 2019a; Huang, 2019; Han, 2020; Liu, 2020; Ma and Zhou, 2020; Meng et al., 2020) had a therapy duration of 3 months (*p* < 0.00001, *I* ^2^ = 88%), one study (Huang et al., 2019b) reported a therapy duration of 4 months, and three studies (Jiang et al., 2018; Wang et al., 2018; Liu, 2019) had a therapy duration of 6 months (*p* < 0.00001, *I* ^2^ = 93%), suggesting some heterogeneity. The random-effect model showed a significant beneficial effect of TCM adjuvant therapy on MMSE scores [MD = 2.55, 95% CI (1.99, 3.10), *p* < 0.00001; MD = 2.53, 95% CI (1.59, 3.47), *p* < 0.00001; MD = 2.91, 95% CI (1.26, 4.56), *p* = 0.0006; MD = 3.11, 95% CI (−0.04, 6.27), *p* = 0.05]. The funnel plot was asymmetrical, indicating the existence of publication bias (Supplementary Figure S3).

### Activities of Daily Living

#### BI

**FIGURE 5:**
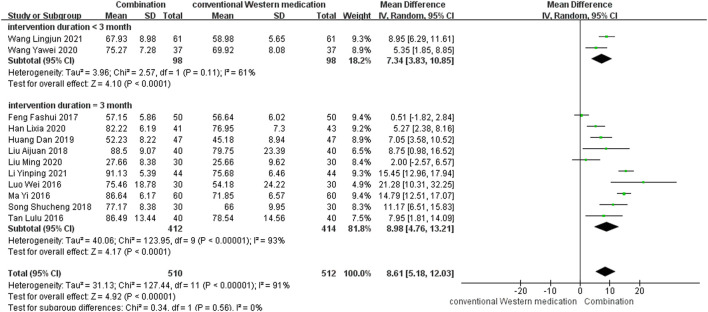
Meta-analysis results for effect of TCM plus conventional Western medication on BI.

TCM plus conventional Western medication was used in 12 studies, as shown in Figure 5. Subgroup analysis was carried out to examine differences in therapy duration. Two study (Wang, 2020b; Wang et al., 2021b) had a therapy duration of <3 months (*p* = 0.11, *I* ^2^ = 61%) and ten studies (Luo and Liu, 2016; Ma and Lin, 2016; Tan et al., 2016; Feng et al., 2017; Liu, 2018; Song, 2018; Huang et al., 2019a; Han, 2020; Liu, 2020; Li, 2021) had a therapy duration of 3 months (*p* < 0.00001, *I* ^2^ = 93%), suggesting significant heterogeneity. The random-effect model showed a significant beneficial effect of TCM adjuvant therapy on BI scores [MD = 7.34, 95% CI (3.83, 10.85), *p* < 0.0001; MD = 8.98, 95% CI (4.76, 13.21), *p* < 0.0001]. The funnel plot was asymmetrical, indicating the existence of publication bias (Supplementary Figure S4).

#### ADL

TCM plus conventional Western medication was used in two studies, as shown in Table 2. The therapy duration was 3 months (Meng et al., 2020) and 4 months (Huang X. R. et al., 2019) respectively, and the results suggest a beneficial effect on ADL scores [MD = −8.64, 95% CI (−9.83, −7.45), *p* < 0.00001; MD = −2.00, 95% CI (−2.94, −1.06), *p* < 0.0001].

**TABLE 2:** Meta-analysis results for effect of TCM plus conventional Western medication on ADL.

Study ID	Intervention duration	Intervention group		Control group		MD [95% CI]
		‾x ± *s*	*n*	‾x ± *s*	*n*	
Meng et al. (2020)	= 3 months	17.31 ± 2.51	42	25.95 ± 3.04	42	−8.64 (−9.83, −7.45)
Huang et al. (2019b)	= 4 months	16 ± 2.675	41	18 ± 1.459	39	−2.00 (−2.94, −1.06)

### Neurological Deficit

#### NIHSS

**FIGURE 6:**
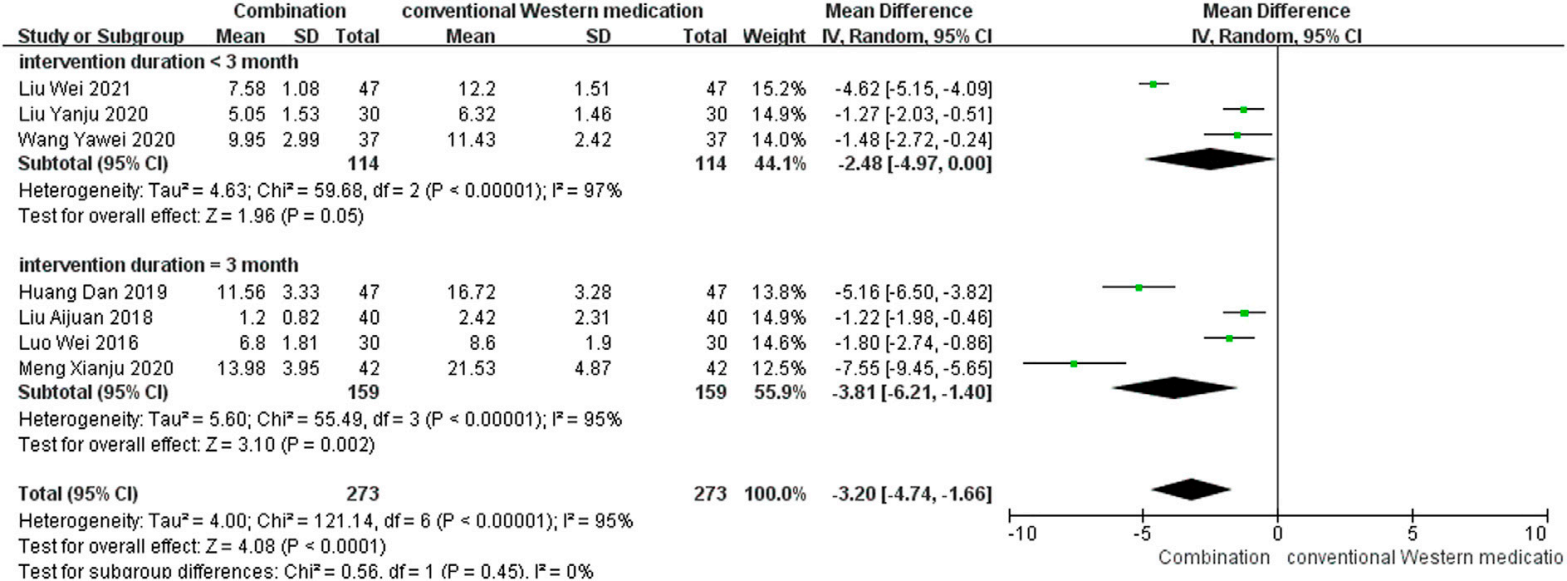
Meta-analysis results for effect of TCM plus conventional Western medication on NIHSS.

TCM plus conventional Western medication was observed in seven studies, as shown in Figure 6. The subgroup analysis of therapy duration showed that three studies (Wang, 2020b; Liu et al., 2020; Liu and Yuan, 2021) had a therapy duration of <3 months (*p* < 0.00001, *I* ^2^ = 97%). Four studies (Luo and Liu, 2016; Liu, 2018; Huang D. et al., 2019a; Meng et al., 2020) had a therapy duration of 3 months (*p* < 0.00001, *I* ^2^ = 95%), suggesting significant heterogeneity. The random-effect model showed that TCM adjuvant therapy significantly improved NIHSS scores [MD = −2.48, 95% CI (−4.97, 0.00), *p* = 0.05; MD = −3.81, 95% CI (−6.21, −1.40), *p* = 0.002].

#### CSS

TCM plus conventional Western medication was used in one study. The therapy duration was 3 months (Song, 2018). The results suggest a significant effect of TCM adjuvant therapy on CSS scores [MD = −2.47, 95% CI (−3.49, −1.45), *p* < 0.00001].

### Adverse Events

**FIGURE 7:**
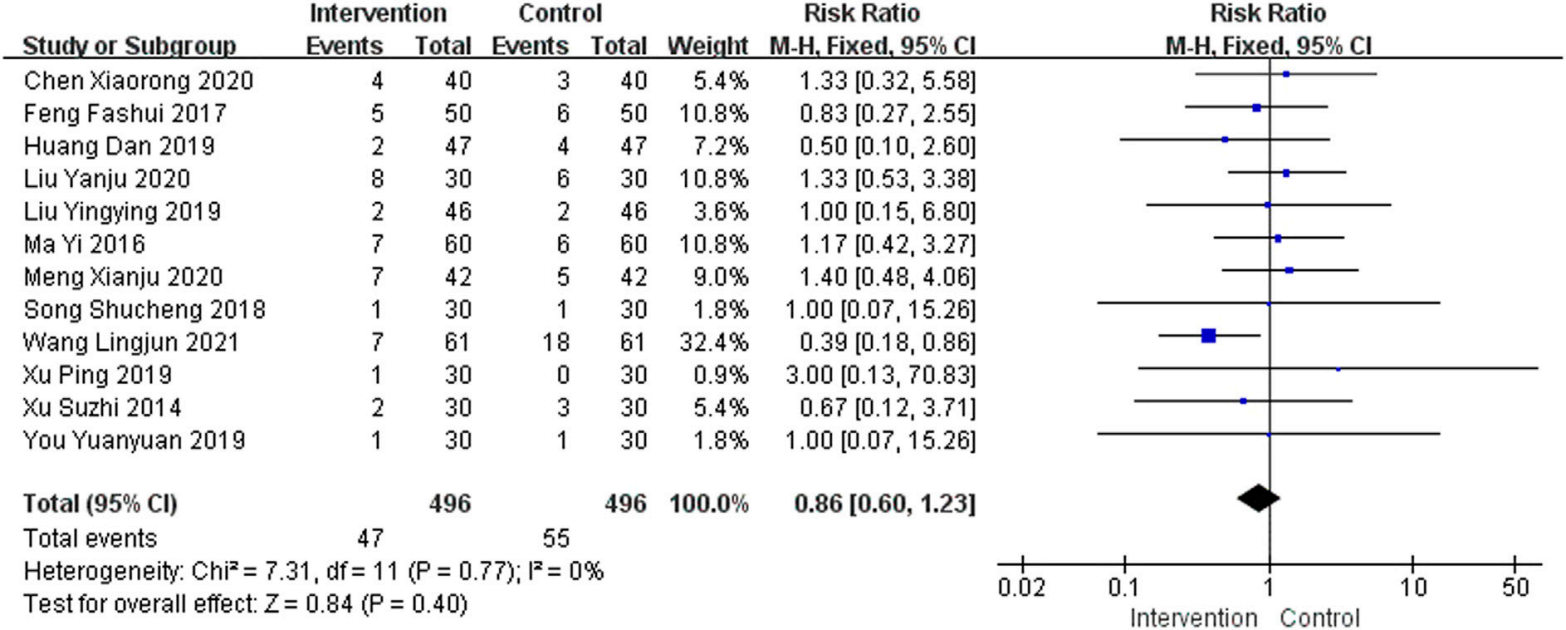
Meta-analysis of adverse events.

A total of 23 studies provided safety-related data. Of these, 11 studies (Cheng et al., 2012; Liu, 2014; Luo and Liu, 2016; Jiang et al., 2018; Wang et al., 2018; Huang et al., 2019b; Huang, 2019; Wang, 2020b; Han, 2020; Liu, 2020; Li, 2021) reported no adverse events, so they were not included in this meta-analysis. A total of 12 studies (Xu et al., 2014; Ma and Lin, 2016; Feng et al., 2017; Song, 2018; Huang et al., 2019a; Liu, 2019; Xu et al., 2019; You, 2019; Chen et al., 2020; Liu et al., 2020; Meng et al., 2020; Wang et al., 2021b) reported gastrointestinal side effects in the intervention group and eight studies (Ma and Lin, 2016; Feng et al., 2017; Huang et al., 2019a; Liu, 2019; Chen et al., 2020; Liu et al., 2020; Meng et al., 2020; Wang et al., 2021b) reported gastrointestinal side effects in the control group, including nausea, vomiting, constipation, stomach discomfort, and loss of appetite. A total of five studies (Ma and Lin, 2016; Feng et al., 2017; Huanget al., 2019a; Chen et al., 2020; Wang et al., 2021b) reported nervous system side effects in the intervention group and six studies (Xu et al., 2014; Huang et al., 2019a; Chen et al., 2020; Liu et al., 2020; Meng et al., 2020; Wang et al., 2021b) reported nervous system side effects in the control group, including drowsiness, insomnia, dizziness, and fatigue. In addition, four studies (Liu, 2019; Chen et al., 2020; Liu et al., 2020; Meng et al., 2020) and six studies (Feng et al., 2017; Song, 2018; Huang et al., 2019a; You, 2019; Chen et al., 2020; Liu et al., 2020) reported dry mouth, frequent urination, rash, blood pressure fluctuations, and other adverse events in the intervention and control groups, respectively. The adverse events were mostly gastrointestinal discomfort, and no serious adverse reactions were found, as shown in Figure 7. The funnel plot was asymmetrical, indicating the existence of publication bias (Supplementary Figure S5).

### GRADE Assessment

GRADE system was used to evaluate the overall evidence of the above six outcomes, as is shown in Table 3. The certainty of evidence indicated very low due to suspected publication bias, significant heterogeneity and serious methodological problems.

**TABLE 3:** Certainty assessment of evidence according to GRADE.

Outcomes	Risk of bias	Inconsistency	Indirectness	Imprecision	Publication bias	No. of patients (studies)	Absolute effects (95% *CI*)	Certainty of the evidence
MoCA	serious^a^	very serious^b^	not serious	not serious	strongly suspected^c^	1,459 (19)	MD 2.87 higher (2.11 higher–3.62 higher)	⊕⃝ ◯ ◯ Very low
MMSE	serious^a^	very serious^b^	not serious	not serious	strongly suspected^c^	2019 (25)	MD 2.56 higher (2 higher–3.12 higher)	⊕⃝ ◯ ◯ Very low
BI	serious^a^	very serious^b^	not serious	not serious	strongly suspected^c^	1,022 (12)	MD 8.61 higher (5.18 higher–12.03 higher)	⊕⃝ ◯ ◯ Very low
ADL	very serious^d^	serious^e^	not serious	not serious	strongly suspected^f^	164 (2)	MD 4.54 lower (5.28 lower–3.8 lower)	⊕⃝ ◯ ◯ Very low
NIHSS	serious^a^	very serious^b^	not serious	not serious	strongly suspected^f^	546 (7)	MD 3.2 lower (4.74 lower–1.66 lower)	⊕⃝ ◯ ◯ Very low
CSS	not serious	serious^e^	not serious	serious^g^	strongly suspected^f^	60 (1)	MD 2.47 lower (3.49 lower–1.45 lower)	⊕⃝ ◯ ◯ Very low

Grade assessment with justification given as follows:

a Most studies are at high RoB.

b I^2^ ≥ 75%.

c Based on the publication bias test, there is apparent asymmetry in the funnel plot.

d All studies are at high RoB.

e Not possible to determine.

f Too few studies.

g Small simple size.

## Discussion

### Summary of Evidence

In this systematic review, we evaluated the clinical efficacy of TCM adjuvant therapy in the treatment of PSCI. A rigorous and reproducible methodology was used to search the literature, and 34 studies involving 2,711 patients with PSCI were identified for analysis. Subgroup analyses of treatment duration showed that TCM adjuvant therapy can promote the recovery of cognitive function, improve activities of daily living, and reduce neurological deficit symptoms in stroke patients. Of the 34 studies analyzed, 11 reported no adverse events related to TCM interventions, and adverse reactions of the remaining studies mainly comprised gastrointestinal discomfort. This indicates that TCM is generally safe and well tolerated for patients with PSCI, which is consistent with previous findings (Shen et al., 2020). Thus, the findings of this meta-analysis suggest, to a limited extent, that TCM adjuvant therapy can be used for PSCI despite methodological flaws in some studies.

### Implications for Practice

The evidence from this systematic review suggests that the use of TCM as an adjuvant therapy may provide additional benefits to PSCI patients, and is generally safe. *Chuanxiong Rhizoma* (Chuanxiong), *Acorita Tatarinowii Rhizoma* (Shichangpu), *Pheretima* (Dilong), and *Angelicae Sinensis Radix* (Danggui) were the herbs most frequently used by the retrieved studies and should be considered a core herbal prescription for PSCI that should be investigated in clinical trials.

### Implications for Research

Considering the clinical efficacy of TCM in the treatment of PSCI, we present the following suggestions for future research.

First, scientific design and rational implementation of RCTs are gold standard for evaluating clinical efficacy of interventions (Schulz et al., 2010; Dwan et al., 2019). However, only one study mentioned double-blinding in study design (You, 2019), which result in uncertainty of research conclusions. Therefore, due to the very low methodological quality of the included studies, future researchers need to improve the quality of RCTs. Prospective registration of trial protocols (e.g., with the Chinese Clinical Trials Registry), adherence to the CONSORT Extension for Chinese Herbal Medicine Formulas 2017 (Cheng et al., 2017) in reporting the results of RCTs of herbal interventions, and use best practices for designing trials of TCMs (e.g., the SPIRIT-TCM Extension 2018 guidelines (Dai et al., 2019) for standardized design) are highly desirable.

Second, some researchers have used ADAS-cog scores to assess improvements in vascular cognitive impairment patients following various treatments (Chan et al., 2018; Kim et al., 2020b). Additionally, some studies have used this scale to estimate cognitive dysfunction after stroke (Falck et al., 2019). However, this scale does not assess vascular factors associated with executive functions, attention, and mental speed (Price et al., 2005; Jokinen et al., 2006). VaDAS-cog is a revised revision of ADAS-cog that includes five additional subtests reflecting vascular conditions (Bastos et al., 2006). VaDAS-cog may be a more sensitive tool with which to assess patients with vascular burden of the brain (Shi et al., 2020). Therefore, the VaDAS-cog or other appropriate outcome measures should be used in future clinical trials to comprehensively evaluate the cognitive status of patients.

Third, PSCI often impairs everyday activities and affects neurological recovery (Hoffmann et al., 2011). Previous studies indicate that PSCI may be accompanied by obvious mental disorders (Zhong et al., 2021), and poor mental status may be associated with higher mortality (Robinson and Jorge, 2016) and affect cognitive recovery (Medeiros et al., 2020). Therefore, more attention needs to be paid to psychological symptoms as well as cognitive function after stroke. However, only one study (Tan et al., 2016) provided Hamilton Rating Scale for Depression scores before and after treatment. More attention should be paid to the psychological status of patients with PSCI. Psychological status in such patients could be evaluated using the Hamilton Rating Scale for Depression, the Hamilton Rating Scale for Anxiety, or other scales commonly used in clinical practice.

Fourth, there is evidence that cognitive function impairment is persistent and dynamic, and has an accelerated downward trend over 6 years after stroke (Levine et al., 2015). In addition, as some patients experienced cognitive decline shortly after stroke and recovered in subsequent few weeks (Nijsse et al., 2017), PSCI can be divided into early-onset PSCI and delayed-onset PSCI, according to the onset time of cognitive impairment (Mok et al., 2017). However, most of the included studies provided insufficient information on the course of disease, the clinical efficacy of TCM could not be objectively evaluated. Therefore, segmental assessment and follow-up of cognitive function should be carried out to determine the best course and the long-term efficacy of TCM in the treatment of PSCI.

Finally, we ranked the frequency of herbal use in the included studies, and summarized the main active components and possible mechanisms for the most common herbs, which is shown in Table 4. The most frequently used herb for PSCI was *Chuanxiong Rhizoma*. Tetramethylpyrazine is the main component of this herb. In terms of neuroprotection, tetramethylpyrazine reduces cognitive impairment by regulating the Janus kinase-signal transducer and activator of transcription signaling pathway, while simultaneously reducing brain edema and blood–brain barrier permeability (Chang et al., 2007; Gong et al., 2019; Huang et al., 2021). The second most frequently used herb was *Acorita Tatarinowii Rhizoma*. The major active ingredients of this herb are α-asarone and β-asarone. These ingredients enhance the proliferation of aberrant neural progenitor cells cultured *in vitro* and their role in neurodegenerative diseases may be mediated by an increase of expression and secretion of neurotrophic factors in astrocytes (Mao et al., 2015; Lam et al., 2019). Antithrombotic protein and enzymes are the most common components of *Pheretima*. The extract of *Pheretima* has excellent anticoagulant and thrombolytic properties, which contribute to the prevention and symptomatic relief of cognitive dysfunction in old age (Ren et al., 2006; Wu et al., 2020). Ligustilide is the main component of *Angelicae Sinensis Radix*. It may improve cognitive dysfunction by reducing mitochondrial dysfunction, generating an antioxidation effect, and restoring synaptic structure (Xin et al., 2013; Li et al., 2015; Xu et al., 2018). As the active components in most TCM formulations and the underlying mechanisms of action remain unclear. More detailed mechanistic studies using modern scientific methods and approaches are needed to elucidate the therapeutic potential mechanisms of TCM for PSCI. Well-designed animal studies and RCTs are also required to validate the physiological and pathological effects of these agents in the treatment of patients with PSCI.

**TABLE 4:** Frequently used herbs and potential mechanisms.

Herbs	Main components	Beneficial effects	Potential mechanisms	Experimental models used
*Chuanxiong Rhizoma* (Chuanxiong)	Tetramethylpyrazine	(1)reduce cognitive impairment	(1) regulate the JAK/STAT signaling pathway	Rats
			(2) increase the expression of tight junction proteins	
		(2) decrease the brain edema and blood-brain barrier permeability	(3) inhibition of HIF-1alpha and TNF-alpha activations	
			(4) inhibition of apoptosis formation	
*Acorita Tatarinowii Rhizoma* (Shichangpu)	α-asarone and β-asarone	(1) promote neurogenesis	(1) enhance the proliferation of Aberrant neural progenitor cells cultured *in vitro*	Rats
		(2) against neurodegeneration and neurodegenerative disorders	(2) stimulate the expression and secretion of neurotrophic factors in astrocytes	
			(3) regulate the PKA signaling pathway	
*Pheretima* (Dilong)	Antithrombotic protein and enzymes	(1) prevention or symptomatic relief of cognitive dysfunction	(1) prolong APTT and decrease fibrinogen content	Pheretima guillelmi; Rats
		(2) anticoagulant and thrombolytic activity		
*Angelicae Sinensis Radix* (Danggui)	Ligustilide	(1) ameliorate cognitive dysfunction	(1) alleviate mitochondrial dysfunction	Rats
			(2) antioxidation effect	
			(3) restore the synaptic structure	

JAK/STAT, Janus kinase-signal transducer and activator of transcription; HIF-1alpha, hypoxia-inducible alpha; TNF-alpha, tumor necrosis factor-alpha; PKA, protein kinase A; APTT, activated partial thromboplastin time.

### Limitations

Some limitations were identified in the primary trials, which need to be addressed in future studies. First, the methodological quality of the included studies was generally low. Although we used a rigorous method to retrieve and select literature, publication bias was inevitable because all the eligible studies were Chinese. Additionally, most studies provided insufficient information on blinding, and 13 of the 34 studies did not provide detailed descriptions of randomization methods, which reduces the credibility of the evidence reported by these studies. Besides, most studies did not use sample size estimation, which undoubtedly increased the risk of exaggerating the intervention effect. Second, the included studies showed clinical heterogeneity. Syndrome differentiation is a unique aspect of TCM (Mei, 2011) and regulates TCM treatment of PSCI (Yao, 2020). However, because of the different symptoms of patients, a large variety of TCMs were used as intervention methods in the included studies, with substantial variation in medication composition, dosage, and treatment duration; this may have led to differences between clinical prescriptions. Third, the use of cognitive evaluation scales needs to be discussed. Most included studies used the MoCA and the MMSE to evaluate the cognitive function of patients. Although the MoCA and MMSE are screening tools (Shi et al., 2018), the PSCI detection rate when using these scales is often lower than the actual incidence (Jokinen et al., 2015). Fourth, treatment duration was not standard across studies. Although most studies assumed an optimum PSCI treatment duration of 6 months (Wang et al., 2021a), most studies used a treatment duration of 0.5–6 months; only three studies had a treatment duration of 6 months. In addition, most eligible studies lacked follow-up information to determine the long-term efficacy of TCM in treating PSCI.

## Conclusion

Despite the significant positive results, the present evidence supports, to a limited extent because of the methodological flaws and herbal heterogeneity, that TCM adjuvant therapy can be used for patients with PSCI. While, further rigorous RCTs are warranted to confirm the efficacy and safety of TCM.

## Data Availability

The original contributions presented in the study are included in the article/Supplementary Material, further inquiries can be directed to the corresponding author.
