# Supplementary material for: Traditional Chinese Medicine for Post-Stroke Cognitive Impairment: A Systematic Review and Meta-Analysis
Source: Front Pharmacol. 2022 Feb 14;13:816333. doi: 10.3389/fphar.2022.816333 (PMC8883343; doi:10.3389/fphar.2022.816333)
Supplement: Supplementary file 1 [file DataSheet1.docx]

**Supplementary Material**

**Table of contents**

[**1 Supplemental tables..............................................................................................................................1**](#_Toc11490)

**1.1 Table S1 The search strategy**[**...............................................................................................1**](#_Toc10609)

**1.2 Table S2 The list of excluded reports.....................................................................................10**

**1.3 Table S3 PRISMA 2020 checklist...........................................................................................14**

**2 Supplemental figures..........................................................................................................................16**

**2.1 Figure S1: Risk of bias summary...........................................................................................16**

**2.2 Figure S2: The funnel plot of MoCA......................................................................................16**

**2.3 Figure S3: The funnel plot of MMSE.....................................................................................17**

**2.4 Figure S4: The funnel plot of BI.............................................................................................17**

**2.5 Figure S5: The funnel plot of Adverse events........................................................................18**

**1 Supplemental tables**

**1.1 Table S1 The search strategy**

| **The search strategy for PubMed** | |
| --- | --- |
| **Nubmer** | **Search terms** |
| #1 | Stroke[MeSH Terms] |
| #2 | Cerebral HemORrhage[MeSH Terms] |
| #3 | ((((((((((((((Stroke*[Title/Abstract]) OR Cerebrovascular Accident*[Title/Abstract]) OR CVA*[Title/Abstract]) OR Apoplexy, Cerebrovascular[Title/Abstract]) OR Vascular Accident*, Brain[Title/Abstract]) OR Cerebrovascular Stroke*[Title/Abstract]) OR Stroke*, Cerebrovascular[Title/Abstract]) OR Apoplexy[Title/Abstract]) OR Cerebral Stroke*[Title/Abstract]) OR Stroke*, Cerebral[Title/Abstract]) OR Stroke*, Acute[Title/Abstract]) OR Acute Stroke*[Title/Abstract]) OR Cerebrovascular Accident*, Acute[Title/Abstract]) OR Acute Cerebrovascular Accident*[Title/Abstract]) |
| #4 | ((((((((HemORrhage*, Cerebrum[Title/Abstract]) OR Cerebrum HemORrhage*[Title/Abstract]) OR Parenchymal HemORrhage*, Cerebral[Title/Abstract]) OR Intracerebral HemORrhage*[Title/Abstract]) OR HemORrhage*, Intracerebral[Title/Abstract]) OR HemORrhage*, Cerebral[Title/Abstract]) OR Cerebral HemORrhage*[Title/Abstract]) OR Brain HemORrhage*, Cerebral[Title/Abstract]) |
| #5 | #1 OR #2 OR #3 OR #4 |
| #6 | Cognitive Dysfunction[MeSH Terms] |
| #7 | ((((((((((((((((((((((((((((Cognitive Dysfunction[Title/Abstract]) OR Cognitive Dysfunctions[Title/Abstract]) OR Dysfunction, Cognitive[Title/Abstract]) OR Dysfunctions, Cognitive[Title/Abstract]) OR Cognitive Impairments[Title/Abstract]) OR Cognitive Impairment[Title/Abstract]) OR Impairment, Cognitive[Title/Abstract]) OR Impairments, Cognitive[Title/Abstract]) OR Mild Cognitive Impairment[Title/Abstract]) OR Cognitive Impairment, Mild[Title/Abstract]) OR Cognitive Impairments, Mild[Title/Abstract]) OR Impairment, Mild Cognitive[Title/Abstract]) OR Impairments, Mild Cognitive[Title/Abstract]) OR Mild Cognitive Impairments[Title/Abstract]) OR Mild Neurocognitive DisORder[Title/Abstract]) OR DisORder, Mild Neurocognitive[Title/Abstract]) OR DisORders, Mild Neurocognitive[Title/Abstract]) OR Mild Neurocognitive DisORders[Title/Abstract]) OR Neurocognitive DisORder, Mild[Title/Abstract]) OR Neurocognitive DisORders[Title/Abstract]) OR Mild Cognitive Decline[Title/Abstract]) OR Cognitive Declines[Title/Abstract]) OR Decline, Cognitive[Title/Abstract]) OR Declines, Cognitive[Title/Abstract]) OR Mental DeteriORation[Title/Abstract]) OR DeteriORation, Mental[Title/Abstract]) OR DeteriORations, Mental[Title/Abstract]) OR Mental DeteriORations[Title/Abstract]) |
| #8 | Dementia, Vascular[MeSH Terms] |
| #9 | ((((((((((((((((((((((((((((((((((((((((Dementia, Vascular[Title/Abstract]) OR Dementias, Vascular[Title/Abstract]) OR Vascular Dementias[Title/Abstract]) OR Vascular Dementia[Title/Abstract]) OR Vascular Dementia, Acute Onset[Title/Abstract]) OR Acute Onset Vascular Dementia[Title/Abstract]) OR SubcORtical Vascular Dementia[Title/Abstract]) OR Dementia, SubcORtical Vascular[Title/Abstract]) OR Dementias, SubcORtical Vascular[Title/Abstract]) OR SubcORtical Vascular Dementias[Title/Abstract]) OR Vascular Dementia, SubcORtical[Title/Abstract]) OR Vascular Dementias, SubcORtical[Title/Abstract]) OR Arteriosclerotic Dementia[Title/Abstract]) OR Arteriosclerotic Dementias[Title/Abstract]) OR Dementia, Arteriosclerotic[Title/Abstract]) OR Dementias, Arteriosclerotic[Title/Abstract]) OR Binswanger Disease[Title/Abstract]) OR Disease, Binswanger[Title/Abstract]) OR Chronic Progressive SubcORtical Encephalopathy[Title/Abstract]) OR Binswanger Encephalopathy[Title/Abstract]) OR Leukoencephalopathy, SubcORtical[Title/Abstract]) OR Leukoencephalopathies, SubcORtical[Title/Abstract]) OR SubcORtical Leukoencephalopathies[Title/Abstract]) OR Encephalopathy, SubcORtical Arteriosclerotic[Title/Abstract]) OR Binswanger's Disease[Title/Abstract]) OR Binswangers Disease[Title/Abstract]) OR Disease, Binswanger's[Title/Abstract]) OR Encephalopathy, SubcORtical, Chronic Progressive[Title/Abstract]) OR SubcORtical Encephalopathy, Chronic Progressive[Title/Abstract]) OR SubcORtical Leukoencephalopathy[Title/Abstract]) OR SubcORtical Arteriosclerotic Encephalopathy[Title/Abstract]) OR Arteriosclerotic Encephalopathy, SubcORtical[Title/Abstract]) OR Arteriosclerotic Encephalopathies, SubcORtical[Title/Abstract]) OR Encephalopathies, SubcORtical Arteriosclerotic[Title/Abstract]) OR SubcORtical Arteriosclerotic Encephalopathies[Title/Abstract]) OR Encephalopathy, Binswanger's[Title/Abstract]) OR Binswanger's Encephalopathy[Title/Abstract]) OR Encephalopathy, Binswangers[Title/Abstract]) OR Encephalopathy, Binswanger[Title/Abstract]) OR Encephalopathy, Chronic Progressive SubcORtical[Title/Abstract]) |
| #10 | Dementia, Multi-Infarct[MeSH Terms] |
| #11 | ((((((((((((((((((Dementia, Multi-Infarct[Title/Abstract]) OR Dementia, Multi Infarct[Title/Abstract]) OR Dementias, Multi-Infarct[Title/Abstract]) OR Multi-Infarct Dementias[Title/Abstract]) OR Dementia Multi-Infarct[Title/Abstract]) OR Dementia Multi Infarct[Title/Abstract]) OR Dementia Multi-Infarcts[Title/Abstract]) OR Multi-Infarct, Dementia[Title/Abstract]) OR Multi-Infarcts, Dementia[Title/Abstract]) OR Dementia, Multiinfarct[Title/Abstract]) OR Dementias, Multiinfarct[Title/Abstract]) OR Multiinfarct Dementia[Title/Abstract]) OR Multiinfarct Dementias[Title/Abstract]) OR Multi-Infarct Dementia[Title/Abstract]) OR Multi Infarct Dementia[Title/Abstract]) OR Lacunar Dementia[Title/Abstract]) OR Dementia, Lacunar[Title/Abstract]) OR Dementias, Lacunar[Title/Abstract]) OR Lacunar Dementias[Title/Abstract] |
| #12 | (((((((PSCI[Title/Abstract]) OR PSD[Title/Abstract]) OR PSCIND[Title/Abstract]) OR post stroke cognitive impairment[Title/Abstract]) OR cognitive impairment after stroke[Title/Abstract]) OR post stroke cognitive impairment no dementia[Title/Abstract]) OR post stroke dementia[Title/Abstract]) |
| #13 | #6 OR #7 OR #8 OR #9 OR #10 OR #11 OR #12 |
| #14 | Medicine, chinese traditional[MeSH Terms] |
| #15 | Herbal Medicine[MeSH Terms] |
| #16 | Medicine, Traditional[MeSH Terms] |
| #17 | ((((((((((((((Medicine, Chinese Traditional[Title/Abstract]) OR (Traditional Chinese Medicine[Title/Abstract])) OR (Chung I Hsueh[Title/Abstract])) OR (Hsueh, Chung I[Title/Abstract])) OR (Traditional Medicine, Chinese[Title/Abstract])) OR (Zhong Yi Xue[Title/Abstract])) OR (Chinese Traditional Medicine[Title/Abstract])) OR (Chinese Medicine, Traditional[Title/Abstract])) OR (Traditional Tongue Diagnosis[Title/Abstract])) OR (Tongue Diagnoses, Traditional[Title/Abstract])) OR (Tongue Diagnosis, Traditional[Title/Abstract])) OR (Traditional Tongue Diagnoses[Title/Abstract])) OR (Traditional Tongue Assessment[Title/Abstract])) OR (Tongue Assessment, Traditional[Title/Abstract])) OR (Traditional Tongue Assessments[Title/Abstract]) |
| #18 | (((((((((((Herbal Medicine[Title/Abstract]) OR (Medicine, Herbal[Title/Abstract])) OR (Hawaiian Herbal Medicine[Title/Abstract])) OR (Hawaiian Herbal Medicines[Title/Abstract])) OR (Herbal Medicine, Hawaiian[Title/Abstract])) OR (Herbal Medicines, Hawaiian[Title/Abstract])) OR (Medicine, Hawaiian Herbal[Title/Abstract])) OR (Medicines, Hawaiian Herbal[Title/Abstract])) OR (La'au Lapa'au[Title/Abstract])) OR (Laau Lapaau[Title/Abstract])) OR (La au Lapa au[Title/Abstract])) OR (Herbalism[Title/Abstract]) |
| #19 | ((((((((((((((((Medicine, Traditional[Title/Abstract]) OR (Traditional Medicine[Title/Abstract])) OR (Home Remedies[Title/Abstract])) OR (Home Remedy[Title/Abstract])) OR (Remedies, Homeziy[Title/Abstract])) OR (Remedy, Home[Title/Abstract])) OR (Medicine, Primitive[Title/Abstract])) OR (Primitive Medicine[Title/Abstract])) OR (Medicine, Folk[Title/Abstract])) OR (Folk Medicine[Title/Abstract])) OR (Medicine, Indigenous[Title/Abstract])) OR (Indigenous Medicine[Title/Abstract])) OR (Folk Remedies[Title/Abstract])) OR (Folk Remedy[Title/Abstract])) OR (Remedies, Folk[Title/Abstract])) OR (Remedy, Folk[Title/Abstract])) OR (Ethnomedicine[Title/Abstract]) |
| #20 | #14 OR #15 OR #16 OR #17 OR #18 OR #19 |
| #21 | #5 AND #13 AND #20 |
| #22 | ((((((((((randomized controlled trial[Publication Type]) OR controlled clinical trial[Publication Type]) OR randomized[Title/Abstract]) OR placebo[Title/Abstract]) OR drug therapy[MeSH Subheading]) OR randomly[Title/Abstract]) OR trial[Title/Abstract]) OR groups[Title/Abstract])) NOT ((animals[MeSH Terms]) NOT humans[MeSH Terms])) |
| #23 | #21 AND #22 |
| **The search strategy for Cochrane library** | |
| **Nubmer** | **Search terms** |
| #1 | MeSH descriptor: [Stroke] explode all trees |
| #2 | (Stroke*):ti,ab,kw OR (Cerebrovascular Accident*):ti,ab,kw OR (CVA*):ti,ab,kw OR (Apoplexy, Cerebrovascular):ti,ab,kw OR (Vascular Accident*, Brain):ti,ab,kw OR (Cerebrovascular Stroke*):ti,ab,kw OR (Stroke*, Cerebrovascular):ti,ab,kw OR (Apoplexy):ti,ab,kw OR (Cerebral Stroke*):ti,ab,kw OR (Stroke*, Cerebral):ti,ab,kw OR (Stroke*, Acute):ti,ab,kw OR (Acute Stroke*):ti,ab,kw OR (Cerebrovascular Accident*, Acute):ti,ab,kw OR (Acute Cerebrovascular Accident*):ti,ab,kw |
| #3 | MeSH descriptor: [Cerebral HemORrhage] explode all trees |
| #4 | (HemORrhage*, Cerebrum):ti,ab,kw OR (Cerebrum HemORrhage*):ti,ab,kw OR (Parenchymal HemORrhage*, Cerebral):ti,ab,kw OR (Intracerebral HemORrhage*):ti,ab,kw OR (HemORrhage*, Intracerebral):ti,ab,kw OR (HemORrhage*, Cerebral):ti,ab,kw OR (Cerebral HemORrhage*):ti,ab,kw OR (Brain HemORrhage*, Cerebral):ti,ab,kw |
| #5 | #1 OR #2 OR #3 OR #4 |
| #6 | MeSH descriptor: [Cognitive Dysfunction] explode all trees |
| #7 | (Dysfunctions, Cognitive):ti,ab,kw OR (Cognitive Impairments):ti,ab,kw OR (Impairments, Cognitive):ti,ab,kw OR (Impairment, Cognitive):ti,ab,kw OR (Dysfunction, Cognitive):ti,ab,kw OR (Cognitive Dysfunctions):ti,ab,kw OR (Cognitive Impairment):ti,ab,kw OR (Mild Neurocognitive DisORders):ti,ab,kw OR (Impairment, Mild Cognitive):ti,ab,kw OR (Neurocognitive DisORders, Mild):ti,ab,kw OR (Mild Cognitive Impairment):ti,ab,kw OR (Impairments, Mild Cognitive):ti,ab,kw OR (DisORder, Mild Neurocognitive):ti,ab,kw OR (Mild Cognitive Impairments):ti,ab,kw OR (Cognitive Impairment, Mild):ti,ab,kw OR (Mild Neurocognitive DisORder):ti,ab,kw OR (DisORders, Mild Neurocognitive):ti,ab,kw OR (Neurocognitive DisORder, Mild):ti,ab,kw OR (Cognitive Impairments, Mild):ti,ab,kw OR (Mental DeteriORations):ti,ab,kw OR (Decline, Cognitive):ti,ab,kw OR (Cognitive Decline):ti,ab,kw OR (Mental DeteriORation):ti,ab,kw OR (DeteriORations, Mental):ti,ab,kw OR (DeteriORation, Mental):ti,ab,kw OR (Declines, Cognitive):ti,ab,kw OR (Cognitive Declines):ti,ab,kw |
| #8 | MeSH descriptor: [Dementia, Vascular] explode all trees |
| #9 | (Vascular Dementias):ti,ab,kw OR (Dementias, Vascular):ti,ab,kw OR (Vascular Dementia):ti,ab,kw OR (Dementia, SubcORtical Vascular):ti,ab,kw OR (Vascular Dementia, SubcORtical):ti,ab,kw OR (Dementias, SubcORtical Vascular):ti,ab,kw OR (SubcORtical Vascular Dementias):ti,ab,kw OR (Vascular Dementias, SubcORtical):ti,ab,kw OR (SubcORtical Vascular Dementia):ti,ab,kw OR (Binswanger's Encephalopathy):ti,ab,kw OR (Binswanger Encephalopathy):ti,ab,kw OR (Chronic Progressive SubcORtical Encephalopathy):ti,ab,kw OR (Encephalopathy, Binswangers):ti,ab,kw OR (Arteriosclerotic Encephalopathies, SubcORtical):ti,ab,kw OR (Disease, Binswanger):ti,ab,kw OR (Arteriosclerotic Encephalopathy, SubcORtical):ti,ab,kw OR (Encephalopathies, SubcORtical Arteriosclerotic):ti,ab,kw OR (SubcORtical Arteriosclerotic Encephalopathies):ti,ab,kw OR (SubcORtical Arteriosclerotic Encephalopathy):ti,ab,kw OR ( Encephalopathy, SubcORtical, Chronic Progressive):ti,ab,kw OR (Leukoencephalopathies, SubcORtical):ti,ab,kw OR (SubcORtical Leukoencephalopathies):ti,ab,kw OR (Encephalopathy, Binswanger's):ti,ab,kw OR (Encephalopathy, Chronic Progressive SubcORtical):ti,ab,kw OR (Disease, Binswanger's):ti,ab,kw OR (Encephalopathy, SubcORtical Arteriosclerotic):ti,ab,kw OR ( Leukoencephalopathy, SubcORtical):ti,ab,kw OR ( SubcORtical Leukoencephalopathy):ti,ab,kw OR (Binswangers Disease):ti,ab,kw OR (SubcORtical Encephalopathy, Chronic Progressive):ti,ab,kw OR (Binswanger's Disease):ti,ab,kw OR (Binswanger Disease):ti,ab,kw OR (Encephalopathy, Binswanger):ti,ab,kw OR (Acute Onset Vascular Dementia):ti,ab,kw OR (Vascular Dementia, Acute Onset):ti,ab,kw OR (Arteriosclerotic Dementias):ti,ab,kw OR (Dementia, Arteriosclerotic):ti,ab,kw OR (Arteriosclerotic Dementia):ti,ab,kw OR (Dementias, Arteriosclerotic):ti,ab,kw |
| #10 | MeSH descriptor: [Dementia, Multi-Infarct] explode all trees |
| #11 | (Multi-Infarct Dementias):ti,ab,kw OR (Multi Infarct Dementia):ti,ab,kw OR (Multi-Infarct, Dementia):ti,ab,kw OR (Multiinfarct Dementia):ti,ab,kw OR (Multi-Infarct Dementia):ti,ab,kw OR (Dementia Multi-Infarcts):ti,ab,kw OR (Dementia, Multi Infarct):ti,ab,kw OR (Dementias, Multi-Infarct):ti,ab,kw OR (Multi-Infarcts, Dementia):ti,ab,kw OR (Dementia, Multiinfarct):ti,ab,kw OR (Dementia Multi-Infarct):ti,ab,kw OR (Dementia Multi Infarct):ti,ab,kw OR (Dementias, Multiinfarct):ti,ab,kw OR (Multiinfarct Dementias):ti,ab,kw OR (Dementia, Lacunar):ti,ab,kw OR (Dementias, Lacunar):ti,ab,kw OR (Lacunar Dementia):ti,ab,kw OR (Lacunar Dementias):ti,ab,kw |
| #12 | (PSCI):ti,ab,kw OR (PSD):ti,ab,kw OR (PSCIND):ti,ab,kw OR (post stroke cognitive impairment):ti,ab,kw OR (cognitive impairment after stroke):ti,ab,kw OR (post stroke cognitive impairment no dementia):ti,ab,kw OR (post stroke dementia):ti,ab,kw |
| #13 | #6 OR #7 OR #8 OR #9 OR #10 OR #11 OR #12 |
| #14 | MeSH descriptor: [Medicine, Chinese Traditional] explode all trees |
| #15 | (Traditional Medicine, Chinese):ti,ab,kw OR (Chinese Traditional Medicine):ti,ab,kw OR (Zhong Yi Xue):ti,ab,kw OR (Chung I Hsueh):ti,ab,kw OR (Hsueh, Chung I):ti,ab,kw OR (Chinese Medicine, Traditional):ti,ab,kw OR (Traditional Chinese Medicine):ti,ab,kw OR (Tongue Diagnosis, Traditional):ti,ab,kw OR (Traditional Tongue Assessments):ti,ab,kw OR (Traditional Tongue Diagnoses):ti,ab,kw OR (Traditional Tongue Assessment):ti,ab,kw OR (Tongue Assessment, Traditional):ti,ab,kw OR (Tongue Diagnoses, Traditional):ti,ab,kw OR (Traditional Tongue Diagnosis):ti,ab,kw |
| #16 | MeSH descriptor: [Herbal Medicine] explode all trees |
| #17 | (Medicine, Herbal):ti,ab,kw OR (La au Lapa au):ti,ab,kw OR (La'au Lapa'au):ti,ab,kw OR (Herbal Medicine, Hawaiian):ti,ab,kw OR (Herbal Medicines, Hawaiian):ti,ab,kw OR (Laau Lapaau):ti,ab,kw OR (Hawaiian Herbal Medicine):ti,ab,kw OR (Hawaiian Herbal Medicines):ti,ab,kw OR (Medicines, Hawaiian Herbal):ti,ab,kw OR (Medicine, Hawaiian Herbal):ti,ab,kw OR (Herbalism):ti,ab,kw |
| #18 | MeSH descriptor: [Medicine, Traditional] explode all trees |
| #19 | (Medicine, Primitive):ti,ab,kw OR (Primitive Medicine):ti,ab,kw OR (Remedies, Home;Remedy, Home):ti,ab,kw OR (Home Remedies):ti,ab,kw OR (Home Remedy):ti,ab,kw OR (Remedy, Folk):ti,ab,kw OR (Remedies, Folk):ti,ab,kw OR (Folk Remedy):ti,ab,kw OR (Folk Remedies):ti,ab,kw OR (Ethnomedicine):ti,ab,kw OR (Indigenous Medicine):ti,ab,kw OR (Medicine, Indigenous):ti,ab,kw OR (Medicine, Folk):ti,ab,kw OR (Folk Medicine):ti,ab,kw OR (Traditional Medicine):ti,ab,kw |
| #20 | #14 OR #15 OR #16 OR #17 OR #18 OR #19 |
| #21 | #5 AND #13 AND #20 |
| **The search strategy for Embase** | |
| **Nubmer** | **Search terms** |
| #1 | 'Stroke'/exp |
| #2 | 'Stroke*':ti,ab OR 'Cerebrovascular Accident':ti,ab OR 'CVA*':ti,ab OR 'Apoplexy, Cerebrovascular':ti,ab OR 'Vascular Accident*, Brain':ti,ab OR 'Cerebrovascular Stroke*':ti,ab OR 'Stroke*, Cerebrovascular':ti,ab OR 'Apoplexy':ti,ab OR 'Cerebral Stroke*':ti,ab OR 'Stroke*, Cerebral':ti,ab OR 'Stroke*, Acute':ti,ab OR 'Acute Stroke*':ti,ab OR 'Cerebrovascular Accident*, Acute':ti,ab OR 'Acute Cerebrovascular Accident*':ti,ab |
| #3 | 'Cerebral HemORrhage'/exp |
| #4 | 'HemORrhage*, Cerebrum':ti,ab OR 'Cerebrum HemORrhage*':ti,ab OR 'Parenchymal HemORrhage*, Cerebral':ti,ab OR 'Intracerebral HemORrhage*':ti,ab OR 'HemORrhage*, Intracerebral':ti,ab OR 'HemORrhage*, Cerebral':ti,ab OR 'Cerebral HemORrhage*':ti,ab OR 'Brain HemORrhage*, Cerebral':ti,ab |
| #5 | #1 OR #2 OR #3 OR #4 |
| #6 | 'Vascular Neurocognitive DisORders':ti,ab OR 'Vascular Cognitive DisORders':ti,ab OR 'Vascular Neurocognitive DisORder':ti,ab OR 'Vascular Mild Cognitive Impairment':ti,ab OR 'Post Stroke Cognitive Impairment':ti,ab OR 'Post-Stroke Dementia':ti,ab OR 'SubcORtical Vascular Dementia':ti,ab OR 'Ischemic Vascular Dementia':ti,ab OR 'SubcORtical Vascular Disease':ti,ab OR 'Primary Degenerative Dementia':ti,ab OR 'Mixed Dementia':ti,ab OR 'Cerebrovascular Cognitive Impairment':ti,ab OR 'Multi Infarct Dementia':ti,ab OR 'SubcORtical Ischemic Vascular Disease':ti,ab |
| #7 | 'Arteriosclerotic Encephalopathies, SubcORtical':ti,ab OR 'Encephalopathies, SubcORtical Arteriosclerotic':ti,ab OR 'SubcORtical Arteriosclerotic Encephalopathies':ti,ab OR 'Encephalopathy,Binswangers':ti,ab OR 'Encephalopathy, Binswanger':ti,ab OR 'Encephalopathy, Chronic Progressive SubcORtical':ti,ab OR 'SubcORtical Ischemie Vasculardementia':ti,ab |
| #8 | 'Encephalopathy, SubcORtical, Chronic Progressive':ti,ab OR 'SubcORtical Encephalopathy, Chronic Progressive':ti,ab OR 'SubcORtical Leukoencephalopathy':ti,ab OR 'SubcORtical Arteriosclerotic Encephalopathy':ti,ab OR 'Arteriosclerotic Encephalopathy, SubcORtical':ti,ab |
| #9 | 'Binswangers Disease':ti,ab OR 'SubcORtical Leukoencephalopathies':ti,ab OR 'Encephalopathy, SubcORtical Arteriosclerotic':ti,ab |
| #10 | 'Binswanger Disease':ti,ab OR 'Disease, Binswanger':ti,ab OR 'Chronic Progressive SubcORtical Encephalopathy':ti,ab OR 'Binswanger Encephalopathy':ti,ab OR 'Leukoencephalopathy, SubcORtical':ti,ab OR 'Leukoencephalopathies, SubcORtical':ti,ab OR 'Vascular Dementias, SubcORtical':ti,ab OR 'Arteriosclerotic Dementia':ti,ab OR 'Arteriosclerotic Dementias':ti,ab OR 'Dementia, Arteriosclerotic':ti,ab OR 'Dementias, Arteriosclerotic':ti,ab |
| #11 | 'Acute Onset Vascular Dementia':ti,ab OR 'SubcORtical Vascular Dementia':ti,ab OR 'Dementia, SubcORtical Vascular':ti,ab OR 'Dementias, SubcORtical Vascular':ti,ab OR 'SubcORtical Vascular Dementias':ti,ab OR 'Vascular Dementia, SubcORtical':ti,ab OR 'Dementias, Vascular':ti,ab OR 'Vascular Dementias':ti,ab OR 'Vascular Dementia':ti,ab OR 'Vascular Dementia, Acute Onset':ti,ab OR 'Lacunar Dementia':ti,ab OR 'Dementia, Lacunar':ti,ab OR 'Dementias, Lacunar':ti,ab OR 'Lacunar Dementias':ti,ab |
| #12 | 'Dementias, Multiinfarct':ti,ab OR 'Multiinfarct Dementia':ti,ab OR 'Multiinfarct Dementias':ti,ab OR 'Multi-Infarct Dementia':ti,ab OR 'Multi Infarct Dementia':ti,ab OR 'Dementia Multi Infarct':ti,ab OR 'Dementia Multi-Infarcts':ti,ab OR 'Multi-Infarct,Dementia':ti,ab OR 'Multi-Infarcts, Dementia':ti,ab OR 'Dementia, Multiinfarct':ti,ab OR 'Dementia, Multi Infarct':ti,ab OR 'Dementias, Multi-Infarct':ti,ab OR 'Multi-Infarct Dementias':ti,ab OR 'Multiinfarct Dementia':ti,ab |
| #13 | 'Multiinfarct Dementia'/exp |
| #14 | 'Cognitive Dysfunction':ti,ab OR 'Cognitive Dysfunctions':ti,ab OR 'Dysfunction, Cognitive':ti,ab OR 'Dysfunctions, Cognitive':ti,ab OR 'Cognitive Impairments':ti,ab OR 'Cognitive Impairment':ti,ab OR 'Impairment, Cognitive':ti,ab OR 'Impairments, Cognitive':ti,ab OR 'Mild Cognitive Impairment':ti,ab OR 'Cognitive Impairment, Mild':ti,ab OR 'Cognitive Impairments, Mild':ti,ab OR 'Impairment, Mild Cognitive':ti,ab OR 'Impairments, Mild Cognitive':ti,ab OR 'Mild Cognitive Impairments':ti,ab OR 'Mild Neurocognitive DisORder':ti,ab OR 'DisORder, Mild Neurocognitive':ti,ab OR 'DisORders, Mild Neurocognitive':ti,ab OR 'Mild Neurocognitive DisORders':ti,ab OR 'Neurocognitive DisORder, Mild':ti,ab OR 'Neurocognitive DisORders, Mild':ti,ab OR 'Cognitive Decline':ti,ab OR 'Cognitive Declines':ti,ab OR 'Decline, Cognitive':ti,ab OR 'Declines, Cognitive':ti,ab OR 'Mental DeteriORation':ti,ab OR 'DeteriORation, Mental':ti,ab OR 'DeteriORations, Mental':ti,ab OR 'Mental DeteriORations':ti,ab |
| #15 | 'PSCI':ti,ab OR 'PSD':ti,ab OR 'PSCIND':ti,ab OR 'post stroke cognitive impairment':ti,ab OR 'cognitive impairment after stroke':ti,ab OR 'post stroke cognitive impairment no dementia':ti,ab OR 'post stroke dementia':ti,ab |
| #16 | #6 OR #7 OR #8 OR #9 OR #10 OR #11 OR #12 OR #13 OR #14 OR #15 |
| #17 | 'Chinese medicine'/exp |
| #18 | 'Chinese herbal medicine':ti,ab OR 'medicine, Chinese traditional':ti,ab OR 'traditional Chinese medicine':ti,ab OR 'Chinese drug':ti,ab OR 'Chinese medicinal fORmulas':ti,ab OR 'fang ji fen lei':ti,ab OR 'fang-ji-fen-lei':ti,ab OR 'fangji fenlei':ti,ab OR 'fangji-fenlei':ti,ab OR 'fangjifenlei':ti,ab OR 'traditional Chinese medicinal fORmula':ti,ab OR 'traditional Chinese medicinal fORmulas':ti,ab OR 'Chinese medicine':ti,ab |
| #19 | 'traditional medicine'/exp |
| #20 | 'ethnomedicine':ti,ab OR 'folk medicine':ti,ab OR 'folk remedy':ti,ab OR 'indigenous medicine':ti,ab OR 'medicine, traditional':ti,ab OR 'native healing':ti,ab OR 'native medicine':ti,ab OR 'traditional healing':ti,ab OR 'traditional indigenous medicine':ti,ab OR 'traditional medicine':ti,ab |
| #21 | 'herbal medicine'/exp |
| #22 | 'botanical medicine':ti,ab OR 'herb medicine':ti,ab OR 'medicine, herbal':ti,ab OR 'medicine, herbal':ti,ab OR 'phyto-medicine':ti,ab OR 'phytomedicine':ti,ab OR 'plant medicine':ti,ab OR 'plant-based medicine':ti,ab OR 'herbal medicine':ti,ab |
| #23 | #17 OR #18 OR #19 OR #20 OR #21 OR #22 |
| #24 | #5 AND #16 AND #23 |
| **The search strategy for CNKI** | |
| (SU = '中风' OR SU = '卒中' OR SU = '脑卒中' OR SU = '脑梗死' OR SU = '脑梗塞' OR SU = '脑栓塞' OR SU = '脑出血' OR SU = '蛛网膜下腔出血') AND (SU = '认知功能' OR SU = '认知障碍' OR SU = '认知受损' OR SU = '认知损伤' OR SU = '痴呆' OR SU = '呆病' OR SU = '呆证' OR SU = '健忘' OR SU = '善忘' OR SU = '痴证' OR SU = '喜忘' OR SU = '白癡' OR SU = '不慧' OR SU = '顡' OR SU = '呆痴' OR SU = '愚痴' OR SU = '神呆' OR SU = '脑髓消' OR SU = '文痴' OR SU = '语言错忘') AND( SU ='中西医' OR SU = '中医' OR SU = '中医药' OR SU = '中药' OR SU = '中成药' OR SU = '中草药' OR SU = '草药' OR SU = '方药' OR SU = '复方' OR SU = '汤' OR SU = '片' OR SU = '饮片' OR SU = '丸' OR SU = '散' OR SU = '胶囊' OR SU = '颗粒' OR SU = '配方颗粒' OR SU = '液' OR SU = '合剂' OR SU = '水' OR SU = '膏' OR SU = '冲剂' OR SU = '丹' OR SU = '酒' OR SU = '茶' OR SU = '露') AND (FT='随机') | |
| **The search strategy for Wanfang** | |
| 主题:((“中西医” OR “中医” OR “中医药” OR “中药” OR “中成药” OR “中草药” OR “草药” OR “方药” OR “复方” OR “汤” OR “片” OR “饮片” OR “丸” OR “散” OR “胶囊” OR “颗粒” OR “配方颗粒” OR “液” OR “合剂” OR “水” OR “膏” OR “冲剂” OR “丹” OR “酒” OR “茶” OR “露”) AND (“中风” OR “卒中” OR “脑卒中” OR “脑梗死” OR “脑梗塞” OR “脑栓塞” OR “脑出血” OR “蛛网膜下腔出血”) AND (“认知功能” OR “认知障碍” OR “认知受损” OR “认知损伤” OR “痴呆” OR “呆病” OR “呆证” OR “健忘” OR “善忘” OR “痴证” OR “喜忘” OR “白癡” OR “不慧” OR “顡” OR “呆痴” OR “愚痴” OR “神呆” OR “脑髓消” OR “文痴” OR “语言错忘”) AND “随机”) | |
| **The search strategy for VIP** | |
| ((M=中西医 OR 中医 OR 中医药 OR 中药 OR 中成药 OR 中草药 OR 草药 OR 方药 OR 复方 OR 汤 OR 片 OR 饮片 OR 丸 OR 散 OR 胶囊 OR 颗粒 OR 配方颗粒 OR 液 OR 合剂 OR 水 OR 膏 OR 冲剂 OR 丹 OR 酒 OR 茶 OR 露) OR (R=中西医 OR 中医 OR 中医药 OR 中药 OR 中成药 OR 中草药 OR 草药 OR 方药 OR 复方 OR 汤 OR 片 OR 饮片 OR 丸 OR 散 OR 胶囊 OR 颗粒 OR 配方颗粒 OR 液 OR 合剂 OR 水 OR 膏 OR 冲剂 OR 丹 OR 酒 OR 茶 OR 露)) AND ((M=中风 OR 卒中 OR 脑卒中 OR 脑梗死 OR 脑梗塞 OR 脑栓塞 OR 脑出血 OR 蛛网膜下腔出血) OR (R=中风 OR 卒中 OR 脑卒中 OR 脑梗死 OR 脑梗塞 OR 脑栓塞 OR 脑出血 OR 蛛网膜下腔出血)) AND ((M=认知功能 OR 认知障碍 OR 认知受损 OR 认知损伤 OR 痴呆 OR 呆病 OR 呆证 OR 健忘 OR 善忘 OR 痴证 OR 喜忘 OR 白癡 OR 不慧 OR 顡 OR 呆痴 OR 愚痴 OR 神呆 OR 脑髓消 OR 文痴 OR 语言错忘) OR (R=认知功能 OR 认知障碍 OR 认知受损 OR 认知损伤 OR 痴呆 OR 呆病 OR 呆证 OR 健忘 OR 善忘 OR 痴证 OR 喜忘 OR 白癡 OR 不慧 OR 顡 OR 呆痴 OR 愚痴 OR 神呆 OR 脑髓消 OR 文痴 OR 语言错忘)) AND ((M=随机) OR (R=随机)) | |
| **The search strategy for SinoMed** | |
| (“中西医”[常用字段:智能] OR “中医”[常用字段:智能] OR "中医药"[常用字段:智能] OR "中药"[常用字段:智能] OR "中成药"[常用字段:智能] OR "中草药"[常用字段:智能] OR "草药"[常用字段:智能] OR "方药"[常用字段:智能] OR "复方"[常用字段:智能] OR "汤"[常用字段:智能] OR "片"[常用字段:智能] OR "饮片"[常用字段:智能] OR "丸"[常用字段:智能] OR "散"[常用字段:智能] OR "胶囊"[常用字段:智能] OR "颗粒"[常用字段:智能] OR "配方颗粒"[常用字段:智能] OR “液”[常用字段:智能] OR “合剂”[常用字段:智能] OR “水”[常用字段:智能] OR “膏”[常用字段:智能] OR “冲剂”[常用字段:智能] OR “丹”[常用字段:智能] OR “酒”[常用字段:智能] OR “茶”[常用字段:智能] OR “露”[常用字段:智能]) AND ("中风"[常用字段:智能] OR "卒中"[常用字段:智能] OR "脑卒中"[常用字段:智能] OR "脑梗死"[常用字段:智能] OR "脑梗塞"[常用字段:智能] OR "脑栓塞"[常用字段:智能] OR "脑出血"[常用字段:智能] OR "蛛网膜下腔出血"[常用字段:智能]) AND ("认知功能"[常用字段:智能] OR "认知障碍"[常用字段:智能] OR "认知受损"[常用字段:智能] OR "认知损伤"[常用字段:智能] OR "痴呆"[常用字段:智能] OR “呆病”[常用字段:智能] OR “呆证”[常用字段:智能] OR “健忘”[常用字段:智能] OR “善忘”[常用字段:智能] OR “痴证”[常用字段:智能] OR “喜忘”[常用字段:智能] OR "白癡"[常用字段:智能] OR "不慧"[常用字段:智能] OR "顡"[常用字段:智能] OR "呆痴"[常用字段:智能] OR “愚痴”[常用字段:智能] OR “神呆”[常用字段:智能] OR “脑髓消”[常用字段:智能] OR “文痴”[常用字段:智能] OR “语言错忘”[常用字段:智能]) AND (随机对照试验[文献类型]) AND (人类[特征词]) | |

**1.2 Table S2 The list of excluded reports**

| **Reports excluded reason** | **Number** |
| --- | --- |
| Not meet diagnostic criteria | 21(Zhao and Guo, 2020; Li, 2020a; Ju, 2019; Yang, 2019; Dai, 2018; Gao, 2018; Lu, 2018; Du and Du, 2018; Guo and Du, 2018; Hong, 2017; Xiong et al., 2017; Yang et al., 2017; Jiang and Li, 2016; Pan et al., 2016b; Wang et al., 2014; Jiang and Xu, 2014; Zhou and Wang, 2013; Chen, 2010; Zhang et al., 2018; Wang, 2015; Zhu et al., 2012) |
| Not meet outcome criteria | 15(Qian, 2020; Li, 2020c; Liu et al., 2020; Wang, 2019; Ge, 2019; Chen, 2019; Di, 2018; Sheng, 2018; Wu et al., 2017; Gao and Li, 2014; Ning et al., 2011; Guo et al., 2020; Liu, 2019; Pan et al., 2016a; Liu, 2020) |
| No full-text | 3(Zhao, 2013; Zhang et al., 2003; Sun, 2020) |
| Duplicate study | 3(Miao et al., 2011; Liu and Wu, 2018; Tan et al., 2016) |
| No clear therapeutic time | 4(Zhang and Zhang, 2019; Zhao, 2017; Wang and Huang, 2016; Li, 2020b) |
| Unclear data | 2(Wei, 2017; Fu, 2014) |

**References**

Chen, X. D. (2010). Effect of Naoxintong capsule combined with Nimodipine on cognitive dysfunction after stroke. Journal of Liaoning University of TCM*.* 12(11), 179-180. doi: CNKI:SUN:LZXB.0.2010-11-085

Chen, Y. (2019). Clinical effect of Compound Changpu Yizhi Decoction combined with Nimodipine in the treatment of mild cognitive impairment after stroke. Capital Food Medicine. 26(3), 175. doi: [10.3969/j.issn.1005-8257.2019.03.158](http://dx.chinadoi.cn/10.3969/j.issn.1005-8257.2019.03.158)

Dai, G. J. (2018). Observation of Therapeutic Effect of Yizhi Qingnao Decoction on Mild Cognitive Dysfunction after Stroke. Chinese Journal of Integrative Medicine on Cardio-/Cerebrovascuiar Disease*.* 16(20), 3048-3051. doi:10.12102/j.issn.1672-1349.2018.20.038

Di, X. Y.(2018). Observation on the effect of integrated traditional Chinese and Western medicine on vascular dementia after cerebral infarction. Cardiovascular Disease Journal of integrated traditional Chinese and Western Medicine*.* 6(25), 149. doi: [10.3969/j.issn.2095-6681.2018.25.110](http://dx.chinadoi.cn/10.3969/j.issn.2095-6681.2018.25.110)

Du, J. H., and Du, M. Z. (2018). Observation of nimodipine, piracetam combined with traditional Chinese medicine and Xiao Luo Luo Ling Decoction in treating vascular dementia after cerebral infarction. Cardiovascular Disease Journal of integrated traditional Chinese and Western Medicine. 6(9), 17-18. doi: [10.3969/j.issn.2095-6681.2018.09.011](http://dx.chinadoi.cn/10.3969/j.issn.2095-6681.2018.09.011)

Fu, H. B. (2014). Effect of integrated Traditional Chinese and Western medicine on vascular dementia after cerebral infarction. Yunnan Journal of Traditional Chinese Medicine and Materia Medica*.* 35(10), 48-49. doi: CNKI:SUN:YZYY.0.2014-10-021

Gao, X., and Li, G. H. (2014). Effect of Buyang Huanwu Decoction on dementia after stroke. Inner Mongolia Journal of Traditional Chinese Medicine. 33(25), 10. doi: [10.3969/j.issn.1006-0979.2014.25.009](http://dx.chinadoi.cn/10.3969/j.issn.1006-0979.2014.25.009)

Gao, Y. T. (2018). Randomized, Parallel Study of DihuangYinzi and Anisocitine in the Treatment of PostStroke Dementia (Kidney Deficiency and Phlegm Stasis). Journal of Practical Traditional Chinese Internal Medicine. 32(7), 43-46. doi:10.13729/j.issn.1671-7813.Z20180006

Ge, Z. H. (2019). Effect of Compound Changpu Yizhi Decoction combined with Nimodipine on cognitive dysfunction after stroke. Health Protection and Promotion. (6), 95-96. doi: 10.3969/j.issn.1671-0223(x).2019.03.066

Guo, T., Jia, Y. B., and Wu, X. Y. (2020). Effect of Huannao Yicong Recipe on the treatment of cognitive dysfunction in elderly patients with stroke. Journal of Baotou Medical College*.* 36(9), 117-120. doi: 10.16833/j.cnki.jbmc.2020.09.033

Guo, Z. H., and Du, Y. N. (2018). Effect of integrated Traditional Chinese and Western medicine on vascular cognitive impairment after cerebral infarction. Journal of Practical Traditional Chinese Medicine. 34(1), 67-68. doi: [10.3969/j.issn.1004-2814.2018.01.050](http://dx.chinadoi.cn/10.3969/j.issn.1004-2814.2018.01.050)

Hong, K. D. (2017). Effect of Tianzhi granule combined with Citicoline on the cognitive function of dementia patients after stroke. Modern Medicine and Health Research*.* 1(4), 54-56. doi: CNKI:SUN:XYJD.0.2017-04-032

Jiang, X. C., and Li, S. (2016). Effect of Self-made Fang with High Dose of Astragali Combined with Oxiracetam Capsules on Higher Brain Functions of Patients with Dementia after Stroke. Journal of New Chinese Medicine. 48(7), 35-37. doi: 10.13457/j.cnki.jncm.2016.07.016

Jiang, Y., and Xu, F. (2014). Clinical observation of Aricept combined with Naoxintong capsule in treatment of vascular dementia after cerebral infarction. Med J West China*.* 26(6), 698-700. doi: [10.3969/j.issn.1672-3511.2014.06.008](http://dx.chinadoi.cn/10.3969/j.issn.1672-3511.2014.06.008)

Ju, Z. Y. (2019). Effect of Yangxue Qingnao granule combined with Oxiracetam on the improvement of cognitive dysfunction in cerebral infarction. Chin J of Public Health Eng*.* 18(6), 906-908. doi: CNKI:SUN:ZGWX.0.2019-06-041

Li, R. R. (2020a). Clinical observation on vascular dementia after stroke treated by integrated Traditional Chinese and Western medicine. China's Naturopathy*.* 28(14), 66-67. doi: [10.19621/j.cnki.11-3555/r.2020.1432](http://dx.chinadoi.cn/10.19621/j.cnki.11-3555/r.2020.1432)

Li, S. L. (2020b). Effect of Yizhi Qingnao Decoction on the Improvement of Cognitive Function in Stroke Patients. Chinese Medicine Modern Distance Education of China*.* 18(10), 81-83. doi: 10.3969/j.issn.1672-2779.2020.10.032

Li, Y. Z. (2020c). Clinical effect of Compound Changpu Yizhi Decoction combined with Nimodipine in the treatment of mild cognitive impairment after stroke. Health For Everyone. (04), 219-220. doi: CNKI:SUN:RRJK.0.2020-04-335

Liu, A. J., and Wu, M. H. (2018). Clinical study of 40 cases of cognitive impairment after ischemic stroke treated with Chinese and Western medicine. Jiangsu Journal of Traditional Chinese Medicine. 50(8), 29-31. doi: 10.3969/j.issn.1672-397X.2018.08.011

Liu, H. X. (2020). Effect of Fengzhenghuichun Tablet combined with Butylphthalide on mild cognitive impairment after acute cerebral infarction. Practical Clinical Journal of Integrated Traditional Chinese and Western Medicine*.* 20(17), 95-96. doi: [10.13638/j.issn.1671-4040.2020.17.048](http://dx.chinadoi.cn/10.13638/j.issn.1671-4040.2020.17.048)

Liu, M. S., Yu, W. X., and Zhu, B. Q. (2020). FeasibiIity Study of Combined Use of Tongqiao Huoxue Decoction and ButyIphthalide Capsule in the Treatment of Post-stroke VascuIar. Chinese and Foreign Medical Research*.* 18(4), 35-36. doi: [10.14033/j.cnki.cfmr.2020.04.015](http://dx.chinadoi.cn/10.14033/j.cnki.cfmr.2020.04.015)

Liu, X. Q. (2019). Effect of Compound Changpu Yizhi Decoction combined with Nimodipine on mild cognitive impairment after stroke. Diet Health. 6(21), 86.

Lu, A. P. (2018). Clinical study of Compound Changpu Yizhi Decoction combined with Nimodipine in the treatment of mild cognitive impairment after stroke. World Latest Medicine Information. 18(70), 153. doi: 10.19613/j.cnki.1671-3141.2018.70.121

Miao, X. L., Zhu, Y. W., Mo, H. H., and Ning, W. M. (2011). Effects of Ziyin Jiannao Tablet on neurological function of patients with mild cognitive impairment after cerebral infarction. Journal of New Chinese Medicine. 43(4), 19-20. doi:10.13457/j.cnki.jncm.2011.04.006

Ning, W. M., Miao, X. L., Mo, H. H., and Chen, J. Y. (2011). The Clinical And Experimental Research On Ziyinjiannao Tablet Early Treating The Cognition Impairment After Cerebral Infarction. Chinese Archives of Traditional Chinese Medicine. 29(8), 1861-1863. doi: 10.13193/j.archtcm.2011.08.159.ningwm.031

Pan, H., Wang, M. J., Gu, J., Ye, L. S., and Bai, X. (2016a). Clinical study on the treatment of vascular dementia caused by cerebral infarction with Xuanfu method. In: The founding meeting of Encephalopathy Branch of China Association for the Promotion of Traditional Chinese Medicine and the first Chinese Medicine Encephalopathy Master Forum and academic exchange conference. Henan kaifeng. pp 347-350.

Pan, Y. X., Chen, J., Zhong, S. S., Pan, M. Q., and Yang, H. (2016b). Effect of Oxiracetam combined with Traditional Chinese medicine on higher brain function in 33 patients with post-stroke dementia. Chinese Journal of Ethnomedicine and Ethnopharmacy*.* 25(11), 107-108. doi: CNKI:SUN:MZMJ.0.2016-11-066

Qian, G. (2020). Effect of Compound Changpu Yizhi Decoction combined with Nimodipine on mild cognitive dysfunction after stroke. Health For Everyone. (12), 74. doi: CNKI:SUN:RRJK.0.2020-12-119

Sheng, Y. J. (2018). Effect of Compound Changpu Yizhi Decoction combined with Nimodipine on mild cognitive impairment after stroke. Chinese Community Doctors*.* 34(1), 100-101. doi: [10.3969/j.issn.1007-614x.2018.1.61](http://dx.chinadoi.cn/10.3969/j.issn.1007-614x.2018.1.61)

Sun, J. B., 2020. Study on the Therapeutic Effect and Mechanism of Yinaokang on Post-stroke Cognitive Impairment No Dementia. Available at: http://www.chictr.org.cn/showproj.aspx?proj=61835.

Tan, L. L., Zeng, P. P., and Liu, T. (2016). Effect of Shuxue Tongmai granule on cognitive dysfunction after stroke. In: The founding meeting of Encephalopathy Branch of China Association for the Promotion of Traditional Chinese Medicine and the first Chinese Medicine Encephalopathy Master Forum and academic exchange conference. Henan kaifeng. pp 462-464.

Wang, J. X. (2015). Clinical observation of Naoxintong capsule combined with Aricept in the treatment of vascular dementia after cerebral infarction. The World Clinical Medicine*.* 9(12), 223.

Wang, J. S., and Huang, Z. X. (2016). Clinical Effect of Buyang Huanwu Decoction Combined with Citicoline Capsule on Vascular Dementia after Cerebral Infarction of Qi Deficiency and Blood Stasis Type. China & Foreign Medical Treatment. 35(35), 7-10. doi: 10.16662/j.cnki.1674-0742.2016.35.007

Wang, Y. Y. (2019). Effect of Tongqiao Huoxue Decoction combined with Nicergoline on cognitive dysfunction after cerebral infarction. Shenzhen Journal of Integrated Traditional Chinese and Western Medicine*.* 29(15), 26-27. doi: 10.16458/j.cnki.1007-0893.2019.15.012

Wang, Y. B., Wang, J. S., and Liu, X. R. (2014). Clinical observation of treating mild cognitive impairment after ischemic stroke with Qiyuan Decoction. Hebei Medical Journal*.* 36(24), 3792-3793. doi: [10.3969/j.issn.1002-7386.2014.24.046](http://dx.chinadoi.cn/10.3969/j.issn.1002-7386.2014.24.046)

Wei, C. B. (2017). Clinical observation of Butylphthalide combined with Naoan capsule in the treatment of mild cognitive impairment after acute lacunar cerebral infarction. Lishizhen Medicine and Materia Medica Research. 28(5), 1146-1148. doi: [10.3969/j.issn.1008-0805.2017.05.045](http://dx.chinadoi.cn/10.3969/j.issn.1008-0805.2017.05.045)

Wu, X. L., Huang, X. B., Wang, N. Q., Chen,Y. J., and Chen, W. Q. (2017). Effect analysis of Naokang Ⅱ combined with aniracetam for patients with vascular cognitive impairment of none dementia after cerebral infarction. Chin J of Cerebrovase Dis*.* 14(5), 230-234. doi: [10.3969/j.issn.1672-5921.2017.05.002](http://dx.chinadoi.cn/10.3969/j.issn.1672-5921.2017.05.002)

Xiong, Y., Jiang, Y. J., and Zhu, Q. F. (2017). Clinical observation on the treatment of infarct vascular dementia with Xixin Decoction and Oxiracetam. Hunan Journal of Traditional Chinese Medicine. 33(6), 50-51. doi: 10.16808/j.cnki.issn1003-7705.2017.06.021

Yang, C. Z. (2019). Effect of Buyang Huanwu Decoction combined with Citicoline on vascular dementia after qi deficiency and blood stasis cerebral infarction. Renowned Doctor. (6), 216-217. doi: CNKI:SUN:MGYI.0.2019-06-208

Yang, L. J., Tian, J. B., Liu, X. F., Cui, Y., Zhao, J. W., and Gu, J. (2017). Clinical Research of Compound Changpu Yizhi Decoction Combined with Nimodipine in the Treatment of Mild Cognitive Impairment after Stroke. China Pharmacy. 28(14), 1938-1941. doi: 10.6039/j.issn.1001-0408.2017.14.18

Zhang, C. Z., Xie, Y. L., and Zhang, L. L. (2003). Clinical observation of 75 cases of dementia with multiple infarction treated by integrated Traditional Chinese and western medicine. Chinese New Medicine*.* 4(2), 107-108.

Zhang, J. L., Duan, F. Y., and Pan, S. S. (2018). Effect of Jiawei Tongluo Yizhi Decoction combined with Oxiracetam on vascular dementia after stroke. J Mod Med Health*.* 34(13), 2054-2056. doi: 10.3969/j.issn.1009-5519.2018.13.039

Zhang, X. H., and Zhang, W. H. (2019). Effect of integrated traditional Chinese and Western medicine on vascular dementia caused by stroke. Chinese Journal of Urban and Rural Enterprise Hygiene*.* 34(9), 153-154. doi: 10.16286/j.1003-5052.2019.09.063

Zhao, L. P. (2017). Effect of Yangxue Qingnao granule combined olasetan in the treatment of cognitive dysfunction in patients with cerebral infarction. Chinese Journal of Practical Nervous Diseases*.* 20(17), 37-39. doi: [10.3969/j.issn.1673-5110.2017.17.012](http://dx.chinadoi.cn/10.3969/j.issn.1673-5110.2017.17.012)

Zhao, Q. X. (2013). Clinical effect of Tianzhi granule on cognitive dysfunction after acute cerebral infarction. Health Way. 12(11), 51. doi: 10.3969/j.issn.1671-8801.2013.11.053

Zhao, Y., and Guo, Y. (2020). Effect of Yangxue Qingnao Granules Combined with Donepezil Hydrochloride in the Treatment of Cognitive Impairment after Stroke. Clinical Journal of Traditional Chinese Medicine. 32(9), 1707-1710. doi: 10.16448/j.cjtcm.2020.0930

Zhou, L. S., and Wang, J. X. (2013). Clinical observation of Aricept combined with Naoxintong capsule in the treatment of vascular dementia after cerebral infarction. Zhejiang JITCW M. 23(12), 981-982. doi: CNKI:SUN:ZJZH.0.2013-12-011

Zhu, L. M., Wang, Z. Y., Wang, X. Y., and Zhou, S. J. (2012). Clinical Study of Huatuozaizao Pill Combined with Donepezil Hydrochloride in Treating Post-stroke Vascular Dementia with Qi Depression and Blood Stasis Syndrom. In: (2nd) National Symposium on stroke Diagnosis and treatment practice. Guangzhou. pp 34-42.

**1.3 Table S3 PRISMA 2020 checklist**

| **Section and Topic** | **Item #** | **Checklist item** | **Locationwhere item is reported** |
| --- | --- | --- | --- |
| **TITLE** | | |  |
| Title | 1 | Identify the report as a systematic review. | 1 |
| **ABSTRACT** | | |  |
| Abstract | 2 | See the PRISMA 2020 for Abstracts checklist. | 1 |
| **INTRODUCTION** | | |  |
| Rationale | 3 | Describe the rationale for the review in the context of existing knowledge. | 1-2 |
| Objectives | 4 | Provide an explicit statement of the objective(s) or question(s) the review addresses. | 2 |
| **METHODS** | | |  |
| Eligibility criteria | 5 | Specify the inclusion and exclusion criteria for the review and how studies were grouped for the syntheses. | 3 |
| Information sources | 6 | Specify all databases, registers, websites, organisations, reference lists and other sources searched or consulted to identify studies. Specify the date when each source was last searched or consulted. | 3 |
| Search strategy | 7 | Present the full search strategies for all databases, registers and websites, including any filters and limits used. | 3+Table S1 |
| Selection process | 8 | Specify the methods used to decide whether a study met the inclusion criteria of the review, including how many reviewers screened each record and each report retrieved, whether they worked independently, and if applicable, details of automation tools used in the process. | 4 |
| Data collection process | 9 | Specify the methods used to collect data from reports, including how many reviewers collected data from each report, whether they worked independently, any processes for obtaining or confirming data from study investigators, and if applicable, details of automation tools used in the process. | 4 |
| Data items | 10a | List and define all outcomes for which data were sought. Specify whether all results that were compatible with each outcome domain in each study were sought (e.g. for all measures, time points, analyses), and if not, the methods used to decide which results to collect. | 4 |
|  | 10b | List and define all other variables for which data were sought (e.g. participant and intervention characteristics, funding sources). Describe any assumptions made about any missing or unclear information. | 4 |
| Study risk of bias assessment | 11 | Specify the methods used to assess risk of bias in the included studies, including details of the tool(s) used, how many reviewers assessed each study and whether they worked independently, and if applicable, details of automation tools used in the process. | 4 |
| Effect measures | 12 | Specify for each outcome the effect measure(s) (e.g. risk ratio, mean difference) used in the synthesis or presentation of results. | 4 |
| Synthesis methods | 13a | Describe the processes used to decide which studies were eligible for each synthesis (e.g. tabulating the study intervention characteristics and comparing against the planned groups for each synthesis (item #5)). | 4 |
|  | 13b | Describe any methods required to prepare the data for presentation or synthesis, such as handling of missing summary statistics, or data conversions. | 4 |
|  | 13c | Describe any methods used to tabulate or visually display results of individual studies and syntheses. | 4 |
|  | 13d | Describe any methods used to synthesize results and provide a rationale for the choice(s). If meta-analysis was performed, describe the model(s), method(s) to identify the presence and extent of statistical heterogeneity, and software package(s) used. | 4 |
|  | 13e | Describe any methods used to explore possible causes of heterogeneity among study results (e.g. subgroup analysis, meta-regression). | 4 |
|  | 13f | Describe any sensitivity analyses conducted to assess robustness of the synthesized results. | 4 |
| Reporting bias assessment | 14 | Describe any methods used to assess risk of bias due to missing results in a synthesis (arising from reporting biases). | 4 |
| Certainty assessment | 15 | Describe any methods used to assess certainty (or confidence) in the body of evidence for an outcome | 4 |

| **Section and Topic** | **Item #** | **Checklist item** | **Location where item is reported** |
| --- | --- | --- | --- |
| **RESULTS** | | |  |
| Study selection | 16a | Describe the results of the search and selection process, from the number of records identified in the search to the number of studies included in the review, ideally using a flow diagram. | 5+Figure 1 |
|  | 16b | Cite studies that might appear to meet the inclusion criteria, but which were excluded, and explain why they were excluded. | Table S2 |
| Study characteristics | 17 | Cite each included study and present its characteristics. | 5+Table 1 |
| Risk of bias in studies | 18 | Present assessments of risk of bias for each included study. | 5+Figure 2+Figure S1 |
| Results of individual studies | 19 | For all outcomes, present, for each study: (a) summary statistics for each group (where appropriate) and (b) an effect estimate and its precision (e.g. confidence/credible interval), ideally using structured tables or plots. | 5-7+ Table 2+Figure 3-7 |
| Results of syntheses | 20a | For each synthesis, briefly summarise the characteristics and risk of bias among contributing studies. | 5-7+ Table 2+Figure 3-7 |
|  | 20b | Present results of all statistical syntheses conducted. If meta-analysis was done, present for each the summary estimate and its precision (e.g. confidence/credible interval) and measures of statistical heterogeneity. If comparing groups, describe the direction of the effect. | 5-7+ Table 2+Figure 3-7 |
|  | 20c | Present results of all investigations of possible causes of heterogeneity among study results. | 5-7+ Table 2+Figure 3-7 |
|  | 20d | Present results of all sensitivity analyses conducted to assess the robustness of the synthesized results. | 5-7+ Table 2+Figure 3-7 |
| Reporting biases | 21 | Present assessments of risk of bias due to missing results (arising from reporting biases) for each synthesis assessed. | 5-7+Figure S2-S5 |
| Certainty of evidence | 22 | Present assessments of certainty (or confidence) in the body of evidence for each outcome assessed. | 7+Table 3 |
| **DISCUSSION** | | |  |
| Discussion | 23a | Provide a general interpretation of the results in the context of other evidence. | 7-8 |
|  | 23b | Discuss any limitations of the evidence included in the review. | 9-10+Table 4 |
|  | 23c | Discuss any limitations of the review processes used. | 9-10+Table 4 |
|  | 23d | Discuss implications of the results for practice, policy, and future research. | 8-9 |
| **OTHER INFORMATION** | | |  |
| Registration and protocol | 24a | Provide registration information for the review, including register name and registration number, or state that the review was not registered. | 2 |
|  | 24b | Indicate where the review protocol can be accessed, or state that a protocol was not prepared. | 2 |
|  | 24c | Describe and explain any amendments to information provided at registration or in the protocol. | 2 |
| Support | 25 | Describe sources of financial or non-financial support for the review, and the role of the funders or sponsors in the review. | 10 |
| Competing interests | 26 | Declare any competing interests of review authors. | 10 |
| Availability of data, code and other materials | 27 | Report which of the following are publicly available and where they can be found: template data collection forms; data extracted from included studies; data used for all analyses; analytic code; any other materials used in the review. | 10 |

*From:* Page MJ, McKenzie JE, Bossuyt PM, Boutron I, Hoffmann TC, Mulrow CD, et al. The PRISMA 2020 statement: an updated guideline for reporting systematic reviews. BMJ 2021;372:n71. doi: 10.1136/bmj.n71For more information, visit: <http://www.prisma-statement.org/>

**2 Supplemental figures**

**2.1 Figure S1: Risk of bias summary**

**
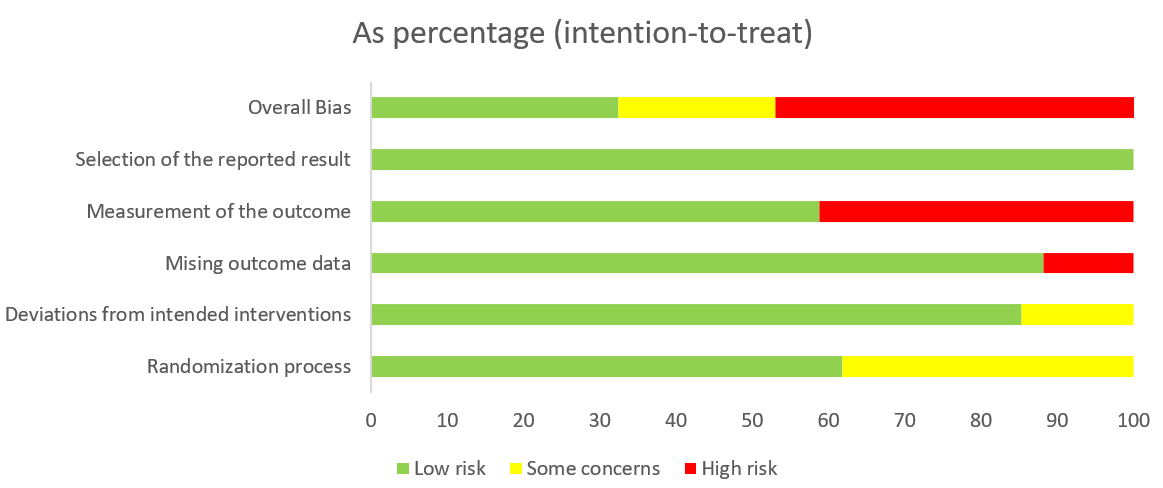
**

**2.2 Figure S2: The funnel plot of MoCA**

**
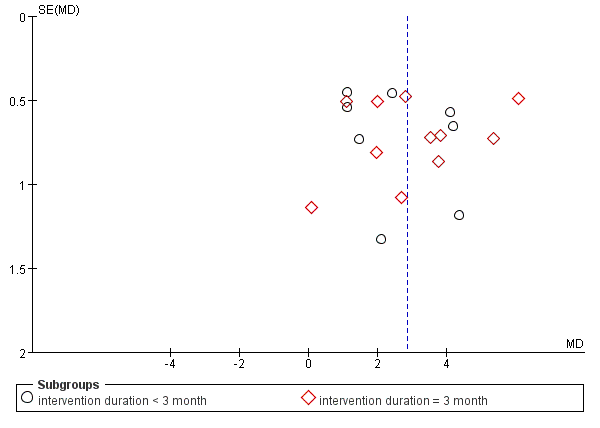
**

**2.3 Figure S3: The funnel plot of MMSE**

**
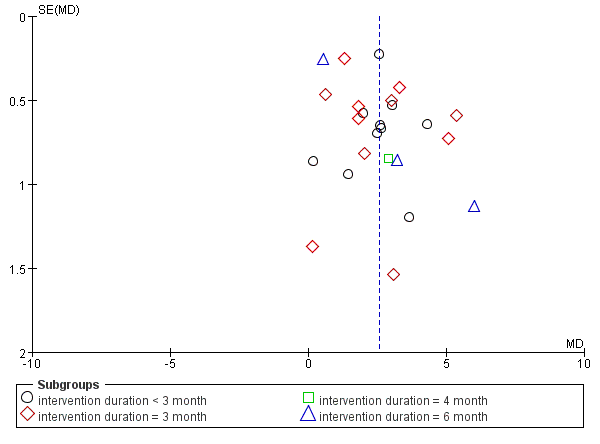
**

**2.4 Figure S4: The funnel plot of BI**

**
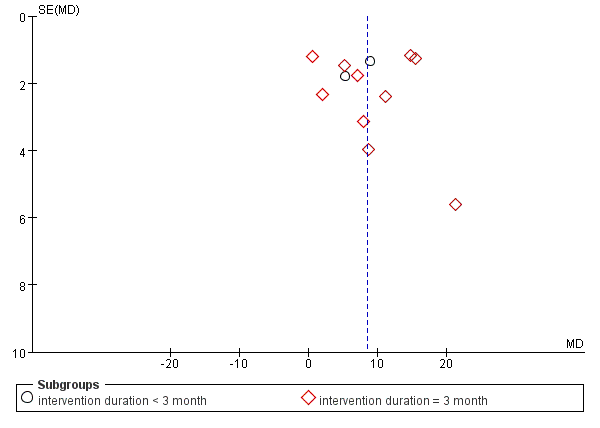
**

**2.5 Figure S5: The funnel plot of Adverse events**

**
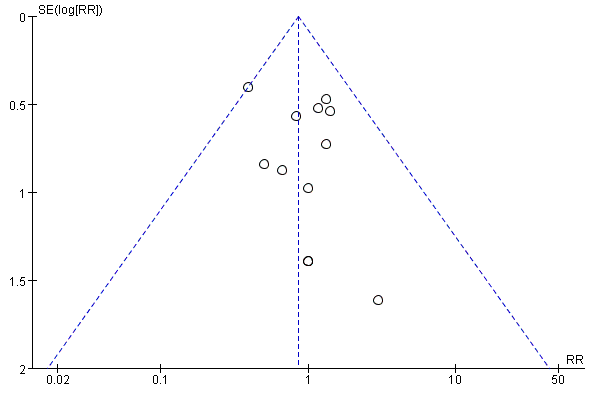
**
